# Chirality-Induced Spin Selectivity in Two-Dimensional Self-Assembled Molecular Networks

**DOI:** 10.1021/jacs.5c12143

**Published:** 2025-11-04

**Authors:** Shammi Rana, Massimiliano Remigio, Lekshmi Aravindan Geetha, Karol Strutyński, Martina Volpi, Sanjay John, Lech Tomasz Baczewski, Yossi Paltiel, Roland Resel, Manuel Melle-Franco, Kunal S. Mali, Yves H. Geerts, Steven De Feyter

**Affiliations:** † Division of Molecular Imaging and Photonics, Department of Chemistry, 54517 KU Leuven, Leuven 3001, Belgium; ‡ KU Leuven Institute for Micro- and Nanoscale Integration (LIMNI), KU Leuven, Celestijnenlaan 200F, Leuven 3001, Belgium; § Laboratoire de Chimie des Polymères, Faculté des Sciences, 26659 Université Libre de Bruxelles (ULB), Boulevard du Triomphe, CP 206/01, Bruxelles 1050, Belgium; ∥ CICECO—Aveiro Institute of Materials, Department of Chemistry, University of Aveiro, Aveiro 3810-193, Portugal; ⊥ Institute of Solid State Physics, 27253 Graz University of Technology, Petersgasse 16, Graz 8010, Austria; # Institute of Physics, Polish Academy of Sciences, Warszawa 02-668, Poland; ∇ Department of Applied Physics, 26742 The Hebrew University, Jerusalem 91904-01, Israel; ○ International Solvay Institutes of Physics and Chemistry, Université Libre de Bruxelles (ULB), CP 231 Boulevard du Triomphe, Bruxelles 1050, Belgium

## Abstract

Chirality-induced spin selectivity (CISS) has been observed in a wide range of helical systems. Here, we report spin-selective electron transport through two-dimensional (2D) self-assembled molecular networks (SAMNs) formed by an enantiopure organic semiconductor with chiral alkyl side chains [dinaphtho­[2,3-b:2‘,3′-f]­thieno­[3,2-*b*]­thiophene (DNTT)] adsorbed on a magnetic substrate with perpendicular anisotropy. Scanning tunneling microscopy and scanning tunneling spectroscopy (STM and STS) were used to directly visualize the molecular arrangement on ferromagnetic surfaces and to measure the spin-dependent electron transport at the solution/solid interface, respectively. A comparison of enantiomorphous SAMNs under identical experimental conditions revealed an enantiospecific magnetic conductance asymmetry (EMA) exceeding 40% at room temperature. These asymmetries were observed when either the molecular enantiomer was changed or the magnetization direction was switched. Our results indicate that the CISS effect is also operative in nonhelical, one-atom-thick systems where the chirality is expressed in 2D, unlocking exciting opportunities for both fundamental research and practical applications.

## Graphical abstract

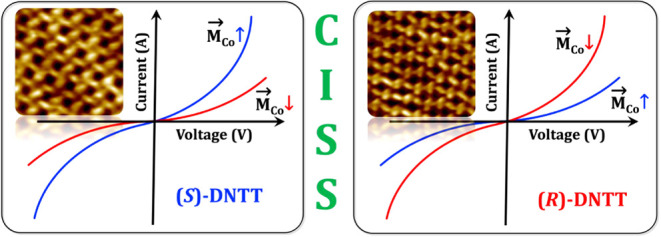

## Introduction

Chirality-induced spin selectivity (CISS) is a process in which chiral molecules preferentially transmit electrons with a specific spin, thereby coupling the spin of the electron to its momentum due to the inherent chirality of the molecule or the material. Electrons passing through a chiral structure are thus “filtered” based on their spin, thereby creating an imbalance between spin-up and spin-down electrons. Such spin polarization (SP) is used to quantify the CISS effect.^1^

The implications of the CISS effect extend across diverse scientific domains, including the design of spintronic devices,^2,3^ chiral separations via homochiral crystallization,^4–6^ and chemical reactions.^7,8^ Due to its influence on spin-selective electron transfer, the CISS effect provides unique insights into processes relevant to biological function, including redox reactions^9^ and allosteric interactions.^10^ It has also been proposed as a plausible explanation for the origin of homochirality in life, suggesting that spin selectivity may have influenced chemical reactions and crystallization processes to favor one chirality, ultimately leading to the emergence of homochirality in biomolecules.^6^

The CISS effect or its different manifestations has been studied experimentally using various techniques. These methods include, but are not limited to, photoemission spectroscopy,^11^ Hall bar measurements,^12^ magneto-optical Kerr effect measurements (spin Seebeck effect),^13^ magnetic circular dichroism,^14^ photoluminescence spectroscopy,^15^ magnetic-conductive probe atomic force microscopy (mC-AFM),^16^ and spin-polarized scanning tunneling microscopy and spectroscopy (STM and STS).^17–19^

Scanning probe methods offer localized measurements of spin-dependent processes and have been essential in understanding how molecular chirality influences spin selectivity.^20^ mC-*AFM* has been the method of choice,^21^ although recent years have witnessed an increased use of STM,^17–19^ given the higher resolution it offers. Spin-polarized STM was recently used to study spin-selective electron transport through single chiral molecules^17^ and the enantioselective adsorption of chiral molecules on magnetic surfaces under ultrahigh vacuum (UHV) conditions.^22^ STM has been employed under ambient conditions to explore various aspects of CISS effects, such as cooperativity, mechanisms, and substrate coupling in polyalanines.^18,19^ While initially discovered in double-stranded DNA,^23^ the CISS effect was later confirmed in various other types of chiral systems, including proteins,^24,25^ peptides,^26^ synthetic polymers,^27^ supramolecular assemblies,^28,29^ bulk crystals,^30,31^ and metal–organic frameworks (MOFs).^32^ Beyond the chirality of individual asymmetric carbon atoms, helical chirality has been the hallmark of systems exhibiting the CISS effect.^17,18,23,33^

This background raises the important question of whether a truly 2D, nonhelical system could also exhibit the CISS effect. This is particularly relevant given the extensive literature on 2D self-assembled molecular networks (SAMNs) that exhibit planar chirality when confined to a solid substrate.^34–36^ Such systems could serve as test beds for studying electron transport across 2D chiral interfaces, facilitated by submolecular resolution STM imaging and highly localized current–voltage (*I*–*V*) measurements.

In this contribution, we report on the chirality-dependent spin filtering by a 2D SAMN formed by dinaphtho­[2,3-*b*:2‘,3′-f]­thieno­[3,2-*b*]­thiophene (**DNTT**, Scheme 1a,b) substituted with chiral side chains. **DNTT** is a member of the organic semiconductor family. It offers high charge mobility, stability, tunability, and easy device integration, making it well-suited for both fundamental research^37^ and spintronic applications.^38^ Recent studies have shown that chiral **DNTT** derivatives exhibit high magnetoresistance in organic field-effect transistors (OFETs).^39^

**Scheme 1:**
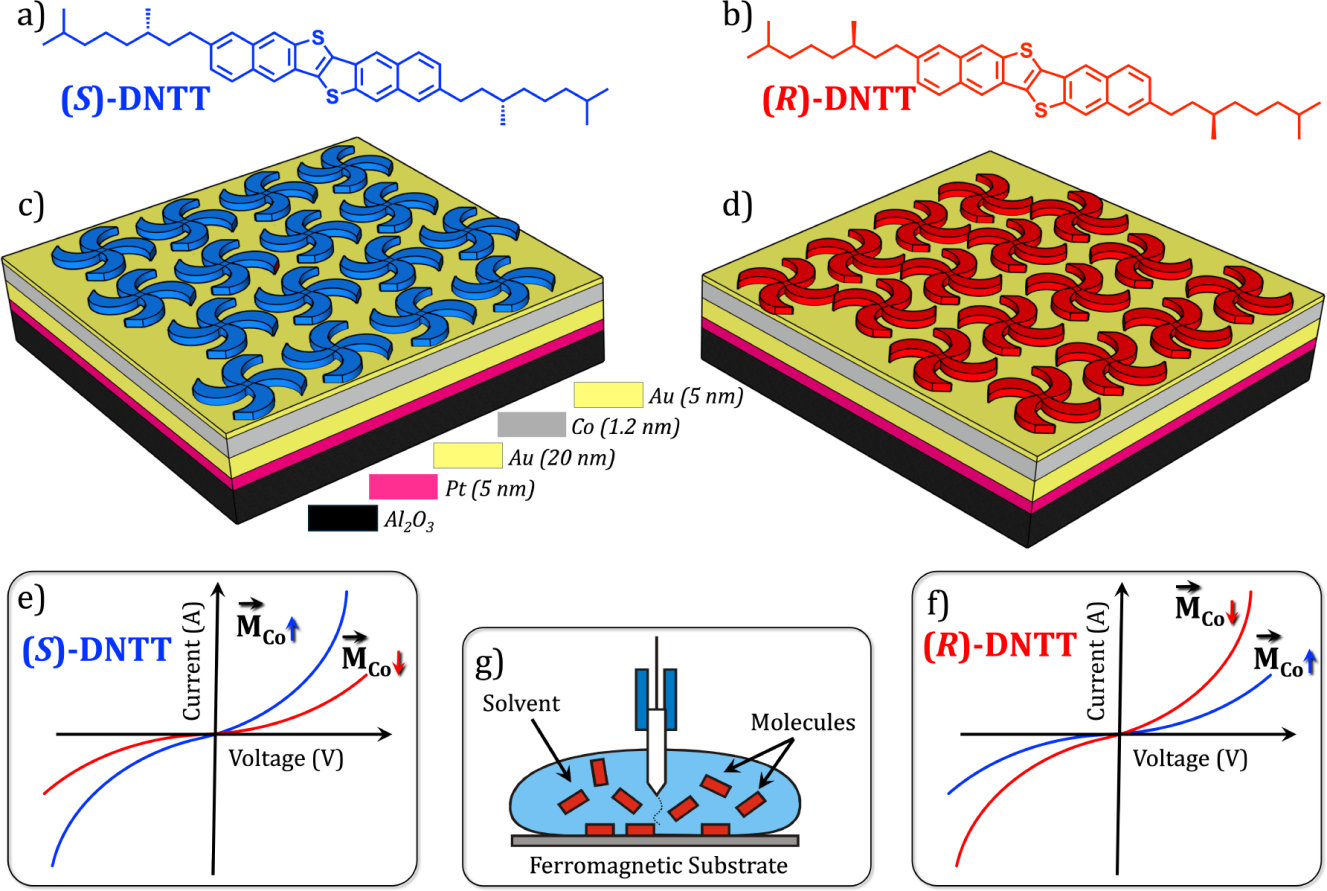
Chirality-Induced Spin Selectivity in the SAMNs of DNTT Derivatives: (a, b) Molecular Structures of **(** ***S*** **)-** and **(** ***R*** **)-DNTT**, Respectively; (c, d) Schematics Showing the Homochiral SAMNs Formed by **(** ***S*** **)-** and **(** ***R*** **)-DNTT** on Au-Coated Ferromagnetic Substrate; (e, f) Schematic Representation of Enantiospecific Magnetic Conductance Asymmetry (EMA) Measurements Carried out on Surfaces Represented in (c) and (d) Using STS with a Non-Magnetic STM Tip at the Solution–Solid Interface (g)

Following this initial study, the SAMNs of enantiopure **DNTT** derivatives formed on epitaxial gold-coated ferromagnetic substrates were characterized using molecular resolution STM, followed by current–voltage (*I*–*V*) measurements. STM data confirmed the formation of homochiral SAMNs (Scheme 1c,d), whereas a comparison of the *I*–*V* data obtained on enantiomorphous SAMNs under identical experimental conditions revealed enantiospecific magnetic conductance asymmetry (EMA) exceeding 40% at room temperature. Notably, these asymmetries emerged upon either switching the molecular enantiomer or reversing the magnetization direction (Scheme 1e,f). Importantly, EMA measurements were performed under wet conditions, highlighting the robustness of the effect in chemically relevant environments. Together, these findings establish a new paradigm for understanding and exploiting the CISS effect in two-dimensional, nonhelical chiral systems, and they open new avenues for investigating CISS-driven processes at electrochemical interfaces.

## Results and Discussion

Scheme 1a and b shows the molecular structures of the chiral **DNTT** derivatives featuring two alkyl side chains derived from citronellol. The SAMNs formed by **(** ***S*** **)-** and **(** ***R*** **)-DNTT** at the 1,2,4-trichlorobenzene (TCB)/ferromagnetic substrate interface were characterized using a nonspin-polarized Pt/Ir (80/20) STM tip. TCB is a commonly used solvent for STM, as well as STS experiments, carried out at the solution–solid interface, and **DNTT** forms stable SAMNs in this solvent. A cobalt thin film (1.2 nm) coated with a 5 nm thick gold layer was selected as the ferromagnetic substrate. The overall substrate structure (Scheme 1c,d), referred to hereafter as a ″ferromagnetic substrate”, consists of epitaxial films of Al_2_O_3_/Pt/Au/Co/Au grown using molecular beam epitaxy (MBE) (see Supporting Information S1.3 for details). The top Au layer serves two purposes: it protects the Co layer from oxidation and provides a chemically defined surface for the physisorption of **DNTT** molecules. The cobalt layer exhibits an out-of-plane easy-axis magnetization direction (perpendicular anisotropy) and a coercive field of ∼11 mT, determined by the polar magneto-optical Kerr effect (P-MOKE) (Figure S1 in the Supporting Information). The out-of-plane magnetization can be readily switched by using an external magnet.

The surface topography of the pristine, as-prepared ferromagnetic substrates was characterized by using STM measurements under ambient conditions. STM images (Figure S2 in the Supporting Information) revealed terraces with diameters ranging from 20 to 50 nm, which are compatible with the minimal requirements for SAMN formation. Initial attempts to form **DNTT** SAMNs were unsuccessful, likely due to contamination from the exposure of the ferromagnetic substrates to ambient conditions (Figure S3 in the Supporting Information). To address this issue, a protocol involving gentle flame-annealing of the gold surface was developed, which enabled SAMN formation. Various controls were performed to assess the ferromagnetic substrate properties after annealing (Figures S4–S7 in the Supporting Information). Surface topography remained unchanged after annealing. X-ray photoelectron spectroscopy (XPS) confirmed that the cobalt layer was not oxidized after annealing, whereas X-ray reflectivity (XRR) measurements showed no interlayer mixing. P-MOKE measurements verified that out-of-plane magnetization was unaffected. The STM data as well as the STS data were obtained by placing a magnet (200–250 mT) underneath the ferromagnetic substrate.

Figure 1a,b shows large-scale STM images of SAMNs of **(** ***S*** **)-DNTT** (Figure 1a) and **(** ***R*** **)-DNTT** (Figure 1b) at the TCB/ferromagnetic substrate interface. Both **(** ***S*** **)-** as well as **(** ***R*** **)-DNTT** form crystalline SAMNs that cover the full surface of the ferromagnetic substrate. A closer look at the small-scale STM images (Figure 1c,d) reveals that the bright rod-shaped features corresponding to the individual **DNTT** units are arranged in a windmill-like tetramer configuration (see also Figure S9 in the Supporting Information). The insets in Figure 1c,d show the digital zooms of individual tetramers. Based on the dimensions of the features observed in STM images, we conclude that the **DNTT** units are adsorbed with their aromatic backbone parallel (face-on) to the surface. The **DNTT** tetramers for the two enantiomers are related by mirror-image symmetry. **(** ***S*** **)-DNTT** exclusively forms tetramers that exhibit clockwise (CW) orientation on the surface, whereas those formed by **(** ***R*** **)-DNTT** are oriented in a counterclockwise (CCW) fashion. The chiral side chains, which determine the orientation of the tetramers, are not visible in the STM images and are located in the darker regions between the tetramers. Within each domain, **DNTT** cores form a crystalline arrangement (unit cell parameters: *a* = 2.5 ± 0.3 nm, *b* = 2.5 ± 0.3 nm, γ = 80 ± 6°, Figure S9). The crystalline arrangement of the chiral **DNTT** units was further corroborated by studying their self-assembly on the Au(111)/mica substrates. Figure 1c,d presents STM images of **DNTT** SAMNs formed at the Au(111)/TCB interface, where the enantiomorphous tetrameric packing is discernible. The unit cell parameters are consistent with those measured for SAMNs on the Au-coated ferromagnetic substrate, further confirming the formation of analogous homochiral SAMNs by enantiopure **DNTT** derivatives.

**Figure 1:**
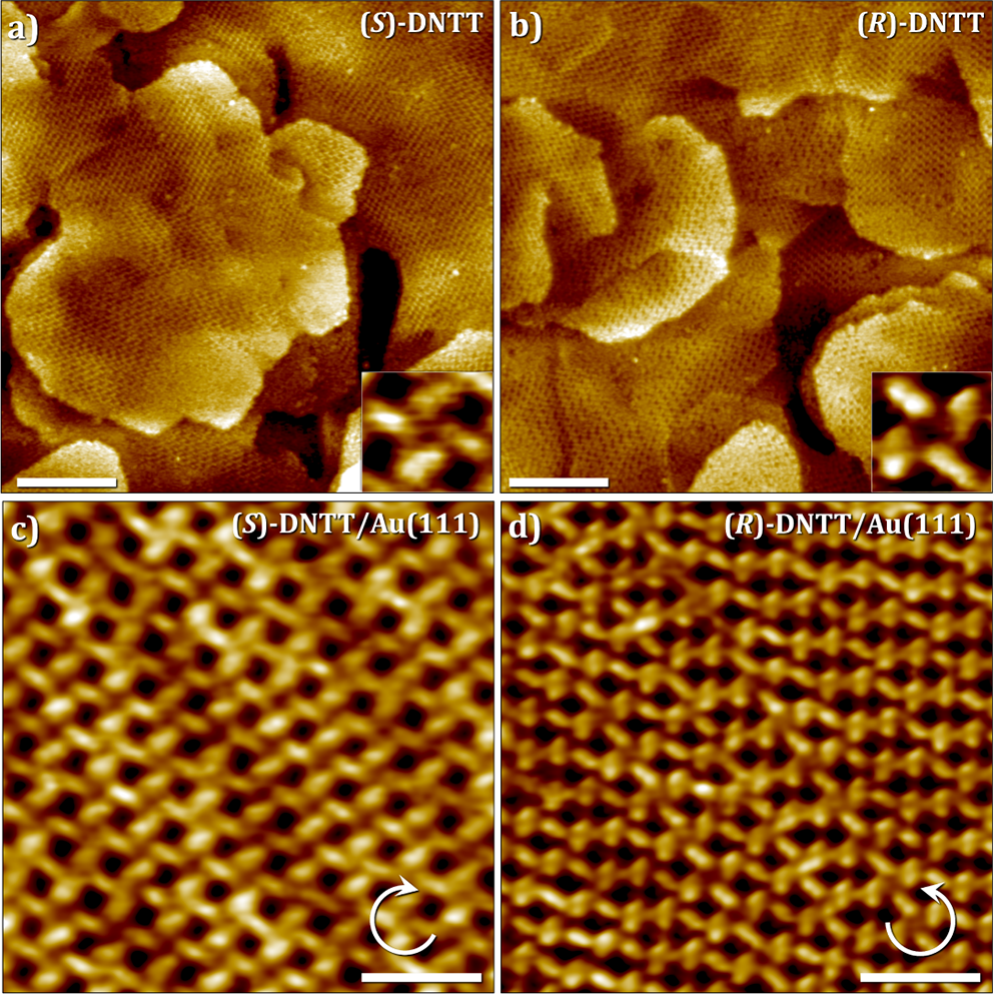
(a, b) Large-scale STM images of the **(** ***S*** **)-** and **(** ***R*** **)-DNTT** SAMNs at the TCB/ferromagnetic substrate interface, respectively. [DNTT] = 8 ´× 10^–6^ M in TCB for both enantiomers. Imaging parameters: (a) *V*_bias_ = −0.20 V, *I*_set_ = 0.10 nA; (b) *V*_bias_ = −0.70 V, *I*_set_ = 0.10 nA. The insets show the representative handedness of the DNTT tetramers formed on the ferromagnetic substrate. Scale bar = 20 nm. (c, d) Small-scale STM images of the **(*S*)**- and **(*R*)-DNTT** SAMN at the TCB/Au(111) interface, respectively. Imaging parameters: (a) *V*_bias_ = −0.7 V, *I*_set_ = 0.10 nA; and (b) *V*_bias_ = −0.4 V, *I*_set_ = 0.03 nA. Scale bar = 5 nm. See Figure S9 in the Supporting Information for additional STM images.

To measure the spin-selective electron transport through these chiral SAMNs, the acquisition of stable, molecular-resolution STM images was followed by the recording of the *I*–*V* curves. The *I*–*V* measurements were conducted in various regions of the samples for both upward and downward magnetization of the Co layer, with all tunneling parameters kept constant throughout. For each magnetization direction, more than 100 individual *I*–*V* curves were recorded. This process was repeated three to five additional times by randomly selecting different positions (separated by a few hundred nanometers), ensuring at least four independent measurements for both **(** ***S*** **)-DNTT** and **(** ***R*** **)-DNTT** (see Figures S10–S13 in Supporting Information).

Figure 2a,b displays the averaged *I*–*V* curves obtained for the SAMNs formed by **(** ***S*** **)-DNTT** and **(** ***R*** **)-DNTT** under opposite magnetization directions of the Co layer. In the case of **(** ***S*** **)-DNTT** SAMN, the magnitude of the current recorded at opposite magnetization directions was found to be different (higher for M↑ compared to M↓), whereas this trend was reversed in the case of SAMN formed by **(** ***R*** **)-DNTT** (higher for M↑ compared to M↓). The differences observed in the *I*–*V* curves obtained on oppositely magnetized ferromagnetic substrates were quantified by calculating EMA^17^ using the following equation:

$$\mathrm{EMA}=\frac{I_{\mathrm{up}}-I_{\mathrm{down}}}{I_{\mathrm{up}}+I_{\mathrm{down}}}\times 100$$

where *I*_up_ and *I*_down_ represent the tunneling currents measured through the same **DNTT** enantiomer but adsorbed on surfaces with opposite out-of-plane magnetization directions. Averaged *I*–*V* curves were used to calculate the EMA values, which are +45 ± 5% for **(** ***S*** **)-DNTT** and −40 ± 5% for **(** ***R*** **)-DNTT** at −1.5 V. The EMAs for the SAMNs of **(** ***S*** **)-DNTT** and **(** ***R*** **)-DNTT** are comparable in magnitude but opposite in sign. This behavior highlights the antisymmetric interaction between the chirality of the molecular units in the SAMN and the magnetization of the ferromagnetic surface, leading to opposite EMAs for the enantiomers while preserving the magnitude of the effect.

**Figure 2:**
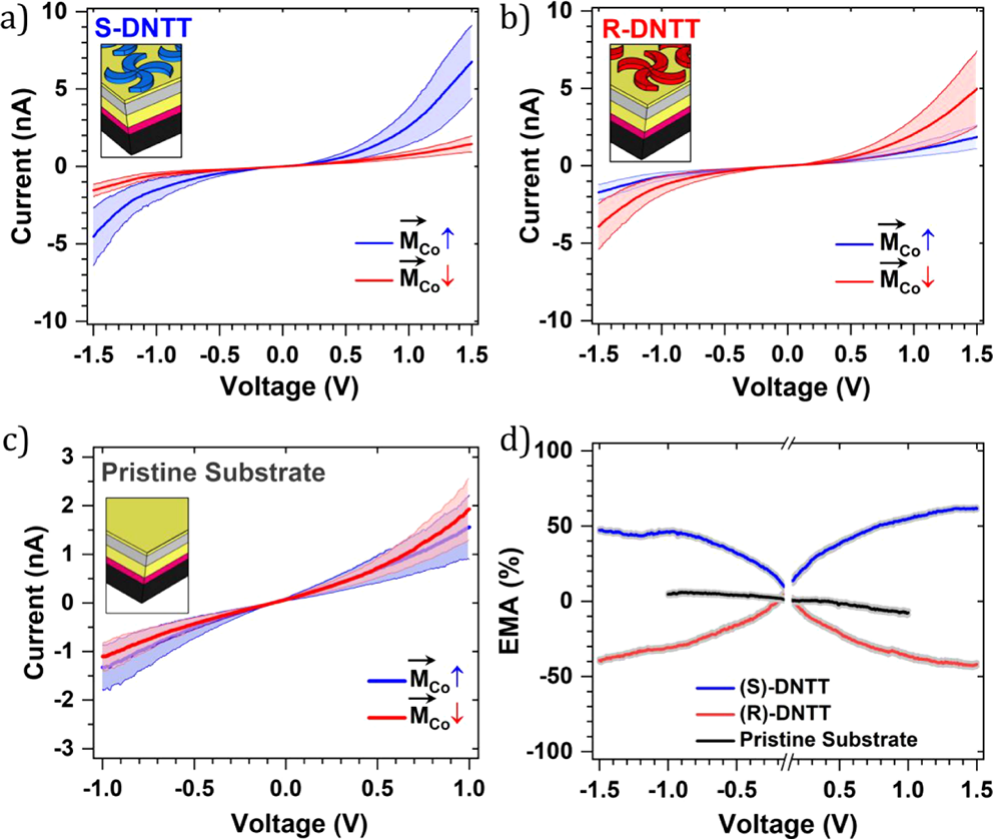
Average *I*–*V* curves acquired for SAMNs of **(*S*)-DNTT** (a), **(*R*)-DNTT** (b), and the pristine ferromagnetic substrate (c) for opposite magnetization directions of the Co layer. Thick blue and red lines show the average, whereas light blue and red areas highlight the standard deviation. Tunneling parameters were kept the same for all *I*–*V* curves; *V*_bias_ = −0.2 V and *I*_set_ = 0.10 nA. Each curve is the average of more than 500 *I*–*V* curves recorded in different areas across the ferromagnetic surface. (d) EMA for **(*S*)-** and **(*R*)-DNTT** SAMNs and the pristine ferromagnetic substrate. The shaded gray area highlights the standard error. See Figures S10–S13 in the Supporting Information for detailed statistics.

The fact that the observed EMA arises primarily due to the CISS effect in electron transport through the chiral SAMNs was further confirmed by carrying out appropriate control experiments. *I*–*V* measurements performed on pristine ferromagnetic substrates at the air/solid interface showed nearly identical current magnitudes for both magnetization directions, resulting in an EMA value of (6 ± 4)% (Figure S14 in the Supporting Information). The voltage range for *I*–*V* curves obtained in air was limited to −1 V to +1 V due to the instability of the system beyond this range, potentially caused by redox processes occurring in the ubiquitous residual water layer that covers surfaces under ambient conditions. EMA from pure TCB solvent on ferromagnetic substrates was found to be around 2–6% at + or −1.5 V (Figure S15 in the Supporting Information). Finally, the role of the ferromagnetic layer was evaluated by conducting *I*–*V* measurements on **(** ***S*** **)-DNTT** SAMN formed on Au(111)/mica substrates in the presence of an external magnet. While pristine Au(111) exhibited small EMA (≈2–6% near ±1.5 V), moderately high (∼20% at −1.5 V and ∼10% at +1.5 V) EMA values were obtained for **(** ***S*** **)-DNTT** SAMN on Au(111) (see Figures S16, S17 in the Supporting Information). The origin of the moderately high EMA observed in the latter case is not entirely clear but likely arises from proximity-induced effects on Au, intrinsic CISS filtering, and experimental asymmetries in the STM junction.

In contrast to previously reported helical systems, the molecules in our system are fully planar and display chirality only through their assembly into tetramers, which adopt mirror-related organizational chirality on an Au-coated ferromagnetic substrate. This organizational chirality is associated with the chiral aliphatic chain coupled to the DNTT core, as reflected in the STM images shown in Figure 1. DFT calculations reveal that models of the assembled prochiral functionalized **DNTT** core can reproduce the observed experimental coverage (Supporting Information Figures S18 to S20). In fact, single **DNTT** units adopt chiral arrangements relative to the symmetry axes of the Au lattice, and the adsorption geometries of the **(** ***S*** **)-** and **(** ***R*** **)-**enantiomers are related by mirror symmetry (Figure 3a and b). To reduce the computational cost, dimethylated-DNTT (**Me-DNTT**) was used for modeling purposes. Although the **Me-DNTT** core itself is not chiral in three dimensions, after adsorption it becomes effectively chiral in 2D. Moreover, DFT calculations indicate that the **DNTT** core donates charge to the Au surface, generating a chiral electrostatic potential pattern at the interface (Figure 3c,d, and Figures S20 and S21 in the Supporting Information). We hypothesize that this asymmetric electronic interaction gives rise to spin–orbit coupling at the molecule–metal interface, potentially enabling spin-dependent electron transport.

**Figure 3:**
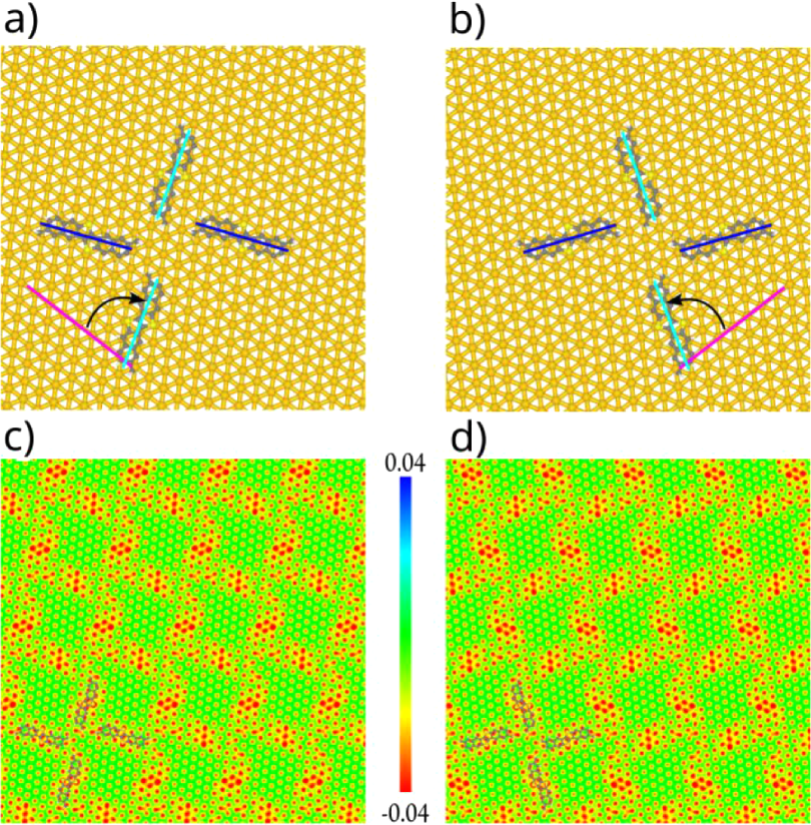
DFT models of **Me-DNTT** tetramer on Au(111) as a proxy for **(** ***S*** **)-DNTT** (a) and the **(** ***R*** **)-DNTT** tetramer (b). Dark and light blue lines highlight the main molecular axis with respect to the gold (110) direction in purple. Chiral electrostatic potential of simulated SAMNs of **Me-DNTT**, as a proxy for **(** ***S*** **)-DNTT** (c) and **(** ***R*** **)-DNTT** (d), and for their packing on the plane between the surface and the molecules.

It is important to note that unlike low-temperature *I*–*V* measurements, where thermal drift has minimal impact on the *X*–*Y* positioning of the STM tip, the measurements reported here rely on averaging the STS data. Consequently, the current recorded across the SAMN represents an average of the currents passing through both the **DNTT** core and the chiral side chains. Thus, it cannot be ascertained whether the observed EMA is due to the point chirality of the side chains, the chiral arrangement of the **DNTT** units, or a combination of both. Therefore, it is reasonable to conclude that in the current system, the observed EMA arises from the organizational chirality of the tetramers, while potential single-molecule contributions from DNTT enantiomers cannot be excluded.

In conclusion, we report an enantiospecific magnetic conductance asymmetry in physisorbed SAMNs. Appreciably high EMA values exceeding 40% were recorded for a molecular system that exhibits a planar adsorption geometry with respect to the substrate surface. The observation of magnetochiral asymmetry in electrical conductance in a system that exhibits 2D chirality bodes well for further investigation into the CISS effect, given the large variety of organic molecular systems that can be studied at the single-molecule level by using scanning probe microscopy under controlled experimental conditions. Furthermore, the emergence of the CISS effect in systems exhibiting 2D chirality, potentially combined with contributions from the point chirality of the side chains, opens up exciting avenues for research into twisted bilayer materials, which exhibit unusual and intriguing physics. These findings add a new dimension to the study of various phenomena currently being studied under the umbrella of the CISS effect.

## Supplementary Material



## Notes

Additional experimental details, characterization of ferromagnetic substrates, a complete collection of *I*–*V* curves, and data from control experiments (PDF)
