# Supplementary material for: Chirality-Induced Spin Selectivity in Two-Dimensional Self-Assembled Molecular Networks
Source: J Am Chem Soc. 2025 Nov 4;147(46):42426–32. doi: 10.1021/jacs.5c12143 (PMC12636013; doi:10.1021/jacs.5c12143)
Supplement: Supplementary file 1 [file ja5c12143_si_001.pdf]

Supporting information

Chirality-Induced Spin Selectivity in Two-Dimensional Self-Assembled Molecular Networks

Shammi Rana,[†,‡] Massimiliano Remigio,[†,‡,#] Lekshmi Aravindan Geetha,[†,‡] Karol Strutyński,[§] Martina Volpi,[#] Sanjay John,[⊥] Lech Tomasz Baczewski,[ψ] Yossi Paltiel,[∇] Roland Resel,[⊥] Manuel Melle Franco[§] Kunal S. Mali,[†,‡]* Yves H. Geerts,[#,Φ]* and Steven De Feyter[†,‡]*

[†] Division of Molecular Imaging and Photonics, Department of Chemistry, KU Leuven, 3001 Leuven, Belgium; [‡] KU Leuven Institute for Micro- and Nanoscale Integration (LIMNI), Celestijnenlaan 200F, KU Leuven, 3001 Leuven, Belgium; [#] Laboratoire de Chimie des Polymères, Faculté des Sciences, Université Libre de Bruxelles (ULB), Boulevard du Triomphe, CP 206/01, Bruxelles 1050, Belgium; [§]CICECO—Aveiro Institute of Materials, Department of Chemistry, University of Aveiro, Aveiro, Portugal[⊥] Institute of Solid State Physics, Graz University of Technology, Petersgasse 16, 8010 Graz, Austria; [ψ] Institute of Physics, Polish Academy of Sciences, Warszawa 02-668, Poland; [∇] Department of Applied Physics, The Hebrew University, Jerusalem, Israel; [Φ] International Solvay Institutes of Physics and Chemistry, Université Libre de Bruxelles (ULB), CP 231 Boulevard du Triomphe, 1050, Bruxelles, Belgium

Contents:

1. S1: Experimental Section
2. S1.1: Synthesis of enantiopure (*R*)-DNTT and (*S*)-DNTT
3. S1.2: STM Measurements
4. S1.3: Growth of ferromagnetic substrates
5. S1.4: Cleaning of Ferromagnetic (Au/Co/Au/Pt/Al_2O_3) substrate
6. S1.5: XPS Measurements
7. S1.6: XRR Measurements
8. S1.7: STS Measurements
9. S1.8: Error bar for STS/EMA measurements
10. S2: Ab-initio Modelling
11. Polar-MOKE characterization of as-prepared ferromagnetic substrates **(Figure S1)**
12. STM images of the pristine ferromagnetic substrate at the air/solid interface **(Figure S2)**
13. STM images of pristine ferromagnetic substrates in the presence of **(*S*)-DNTT** solution in TCB (**Figure S3)**
14. STM characterization of the flame-annealed ferromagnetic substrate at the air/solid interface **(Figure S4)**
15. XPS survey spectrum of the flame-annealed ferromagnetic substrate **(Figure S5)**
16. XRR spectrum of flame-annealed ferromagnetic substrate **(Figure S6)**
17. Comparison of P-MOKE of as-prepared and flame-annealed ferromagnetic substrates **(Figure S7)**
18. STM characterization of flame-annealed Au(111) substrate at the solid/air interface **(Figure S8)**
19. STM images of **(*S*)-DNTT** and **(*R*)-DNTT** at the TCB/ferromagnetic substrate interface **(Figure S9)**
20. STS curves of **(*S*)-DNTT** SAMNs on ferromagnetic substrate for upward magnetization **(Figure S10)**
21. STS curves of **(*S*)-DNTT** on ferromagnetic substrate for downward magnetization **(Figure S11)**
22. STS curves of **(*R*)-DNTT** SAMNs on ferromagnetic substrate for upward magnetization **(Figure S12)**
23. STS curves of **(*R*)-DNTT** SAMNs on ferromagnetic substrate for downward magnetization **(Figure S13)**
24. STS of the pristine ferromagnetic substrate at the air-solid (ferromagnetic substrate) interface **(Figure S14)**
25. STS of the pristine ferromagnetic substrate at the TCB/ferromagnetic substrate interface **(Figure S15)**
26. STS curves on pristine Au(111)/Mica substrate at the solid/air interface **(Figure S16)**
27. STS curves on SAMNs of **(*S*)-DNTT** on Au(111) surface at the solution/solid interface **(Figure S17)**
28. Gold surface used in ab-initio modelling **(Figure S18)**
29. Models of **dimethylated-DNTT** on a gold surface along equivalent rotation **(Figure S19)**
30. Model of **dimethylated-DNTT** SAMN and its electrostatic potential **(Figure S20)**

31. Electrostatic potential of modelled SAMN of **dimethylated-DNTT (Figure S21)**

S1: Experimental Section

S1.1: Synthesis of enantiopure (*R*)-DNTT and (*S*)-DNTT: The DNTT derivatives with chiral side chains were synthesized following the literature report by one of us.[1]

S1.2: STM Measurements

All STM experiments were conducted at the solution/solid interface at room temperature (~22°C). Au-coated (300 nm) mica substrates were purchased from Georg-Albert-PVD, Germany. The Au-coated ferromagnetic substrates were grown using the method described in section **S1.3** (see below). The Au and Au-coated ferromagnetic substrates were stored in a nitrogen box to avoid surface contamination from the surrounding environment. Before every STM experiment, the Au surface was cleaned by flame-annealing using a butane torch. To make a solution of **DNTT** derivatives, 1,2,4-trichlorobenzene (TCB) was used as a solvent. TCB was purchased from Sigma-Aldrich ≥ 99%, and it was used as received without further purification. A few mg of solid powder of **(*S*)-DNTT** or **(*R*)-DNTT** was added to TCB, and then it was sonicated for approximately 45 minutes to make a clear solution. The 8 μM solution (5-10 μl) of **(*R*)-DNTT** or **(*S*)-DNTT** was drop cast onto a flame-annealed Au(111)/mica surface or Au-coated ferromagnetic substrates. Tips for STM measurements were prepared by mechanically cutting a platinum/iridium (Pt/Ir: 80/20%, 0.25 mm in diameter) wire. All STM measurements were performed in constant-current mode. The imaging parameters for each image (main text as well as supporting information) are reported in the figure captions. STM images were processed using Scanning Probe Image Processor (SPIP) software (Image Metrology ApS).

S1.3: Growth of ferromagnetic substrates

The ferromagnetic substrate used in the STM experiments was an epitaxial nanostructure grown by molecular beam epitaxy (MBE). It consisted of sequentially deposited layers, platinum (Pt) buffer layer, gold (Au) buffer, cobalt (Co) layer, and Au cap layer grown on a monocrystalline sapphire Al_2O_3 (0001)) substrate. The exact layer structure was Al_2O_3/Pt (5nm)/Au (20 nm)/Co (1.2 nm)/Au (5 nm). The 5 nm-thick Au(111) top layer served two purposes: protecting the Co layer from oxidation and providing a chemically defined surface for the strong physisorption of **(*S*)-DNTT** or **(*R*)-DNTT** molecules. The 1.2 nm-thick Co layer exhibited out-of-plane easy axis of magnetization (perpendicular anisotropy)

The deposition of layers was carefully controlled, with growth rates kept below 0.1 Å/s. The Pt film was deposited using electron beam evaporation, while Au and Co layers were deposited using effusion cells. To ensure a high-quality surface, the Pt buffer was deposited at 700 °C, and the subsequent Au layer was annealed at 200 °C for 2 hours. The Co layer and Au capping layer were deposited at room temperature

to prevent intermixing at the interfaces. This optimized process produced 20–50 nm-wide atomically flat Au(111) terraces, which are well-suited for the ordered adsorption of **DNTT** molecules.

S1.4: Cleaning of Ferromagnetic (Au/Co/Au/Pt/Al_2O_3) substrate: To clean the ferromagnetic substrate, it was exposed to a low-intensity butane flame for (10-15) seconds. The flame size was kept small, and the flame was passed quickly over the surface to prevent any damage to the surface or inter-layer diffusion. It was checked by P-MOKE that the flame annealing did not alter the magnetic properties of the nanostructure.

S1.5: XPS Measurements: To check the structural integrity of the Cobalt layer in the ferromagnetic substrate during flame-annealing, XPS and XRR measurements were performed**.** XPS measurements were performed with Specs XPS, having an X-ray source Mg (1253.64 eV). A survey scan was taken for the sample as a first step towards the XPS analysis. Since the sample was found to be covered with a Carbon and H_2O contamination layer (O1s peak at 532eV), we could not observe any strong signals from Cobalt. The sample was sputtered (2kV, $5x10^{-7}$mbar) for 5 minutes, and a survey scan was performed after every cycle of sputtering. Depth profiling was carried out for the sample. After 30 minutes of sputtering, a good signal from the Co layer was observed.

From the survey scan obtained after 30 minutes, we can conclude that the sample contains Cobalt, Gold, Carbon, and Oxygen. A high-resolution scan was performed for Co2p and O1s peaks. Upon close examination of the high-resolution scan of Co, the observed binding energy of Co2p3/2 is 778.11 eV, which indicates metallic cobalt.[2-4] There might be some amount of oxidized cobalt. However, this is expected to be negligible. Satellite peak is a characteristic of Co2p3/2.[2-4] A high-resolution scan was performed for the O1s peak. The peak position of O1s (B.E. = 530.3 eV) shows the presence of metallic oxide. However, the percentage of Co-O is <5% compared to that of metallic cobalt. It is worth noting that the sample exhibits the presence of carbon. This carbon may originate from the carbon tape used for grounding the sample during the XPS measurement. Furthermore, there is a possibility that carbon tape may contain oxygen as well.

Overall, the analysis of the Cobalt layer has revealed that it is primarily in a metallic state, with a minimal fraction of oxidized Cobalt, accounting for less than 5% of the total Cobalt content.

S1.6: XRR Measurements: We have performed XRR measurements on flame annealed ferromagnetic substrates (Au/Co/Au/Pt/Al_2O_3) to check the integrity of the Co layer. X-Ray Reflectometry (XRR) analysis is used to measure the thickness, density, and roughness of films. X-ray reflection intensity curves from grazing incident X-ray beams were analyzed to determine thin-film parameters of ferromagnetic substrates, such as thickness, density, and surface roughness. We did not notice any changes in the curves after the flame annealing.

S1.7: STS Measurements: Current-Voltage (I-V) measurements were performed at the solution/solid interface. Before performing I-V measurements on SAMNs of **(*S*)-DNTT** or **(*R*)-DNTT**, stable STM images were acquired with minimal thermal drift, and then only I-V curves were measured. During STS measurements, all tunneling parameters were kept constant. The bias voltage was kept at -0.2 V and the set point at 0.10 nA. For each sample, I-V curves were recorded in multiple areas, separated by a few 100 nm.

S1.8: Error Bar Calculations for STS Curves: Every measurement contains repeated I-Vs. The measurements were obtained keeping the bias constant, which means that every point has the same bias.

1) Mean for every voltage value

$$\bar{I} = \frac{1}{N}\sum_{i=1}^{N} I_i$$

N = number of measurements (I-Vs)

I_i = individual current measurement at a voltage

2) Standard Deviation for voltage value

$$s = \sqrt{\frac{1}{N-1}\sum_{i=1}^{N}(I_i - \bar{I})^2}$$

The sample standard deviation (s) of the valid current measurements at the voltage point.

3) Standard error of the mean for every voltage point

$$\mathrm{SE}(\bar{I}) = \frac{s}{\sqrt{N}}$$

The standard error of mean was calculated for every sample and defined as SE_{up} for the sample measured with the magnetic field up, SE_{down} for the sample measured with the magnetic field down.

4) Propagated error EMA

$$\mathrm{SE}_{\mathrm{EMA}} = \frac{2\sqrt{I_{\mathrm{down}}^2\,\mathrm{SE}_{\mathrm{up}}^2 + I_{\mathrm{up}}^2\,\mathrm{SE}_{\mathrm{down}}^2}}{(I_{\mathrm{up}} + I_{\mathrm{down}})^2}$$

I_{up} = Averaged current from the sample measured with the magnetic field up.

I_{down} = Averaged current from the sample measured with the magnetic field down.

SE_{EMA} is the *standard error* (an uncertainty estimate) of the EMA value. EMA itself is a dimensionless normalized difference between two averaged currents.

The standard error quantifies how uncertain that EMA number is, *given* the uncertainties you have on the two averaged currents I_{up} and I_{down}.

To process I-V data and EMA plots we used python which is available in following link:

STM_IVs_Processor/EMA processor at main · massimilianoremigio97-cmd/STM_IVs_Processor · GitHub

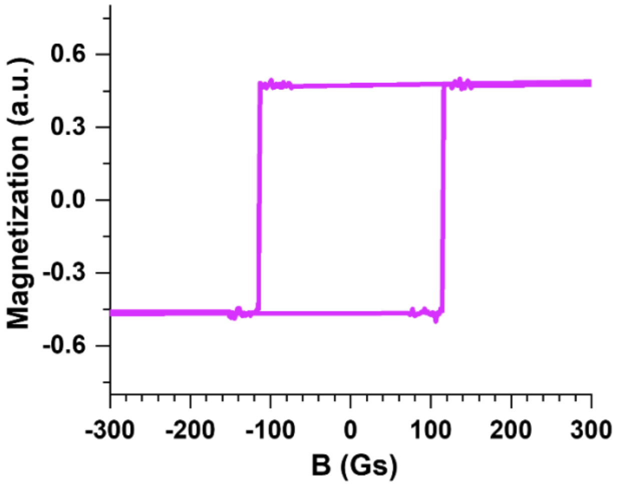

Figure S1: Magnetization curve of the pristine Au/Co/Au/Pt/Al_2O_3 substrate as a function of a magnetic field applied perpendicularly to the sample surface as measured by P-MOKE. The square shape of the loop with a remanence of about 1 proves the existence of a strong perpendicular anisotropy.

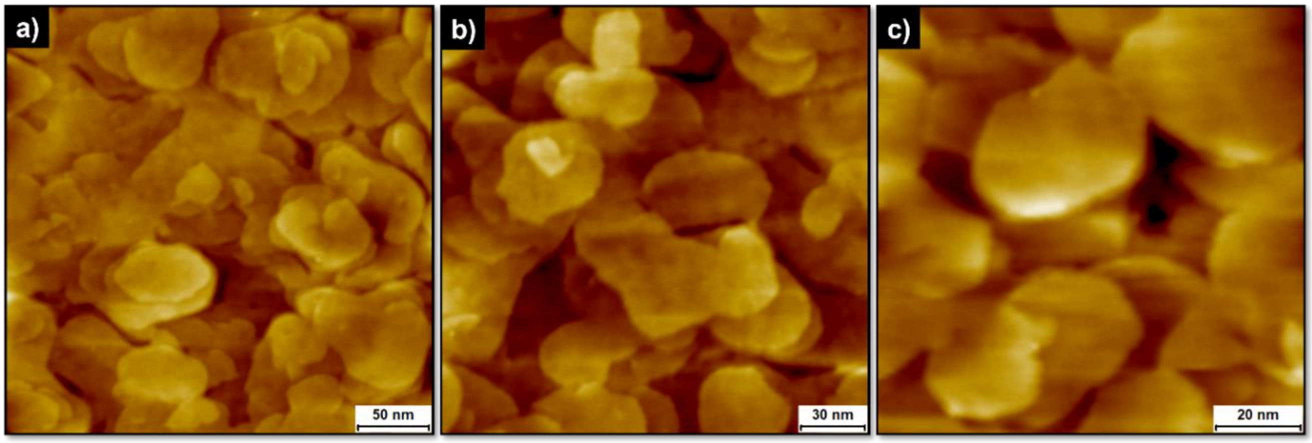

Figure S2: STM images of pristine ferromagnetic (Au/Co/Au/Pt/Al_2O_3) substrate at the air/solid interface. Terrace size varies from 20 – 50 nm. Imaging conditions: (a) V_{bias} = −0.3 V, I_{set} = 0.10 nA; (b) V_{bias} = −0.2 V, I_{set} = 0.1 nA; and (c) V_{bias} = −0.7 V, I_{set} = 0.10 nA.

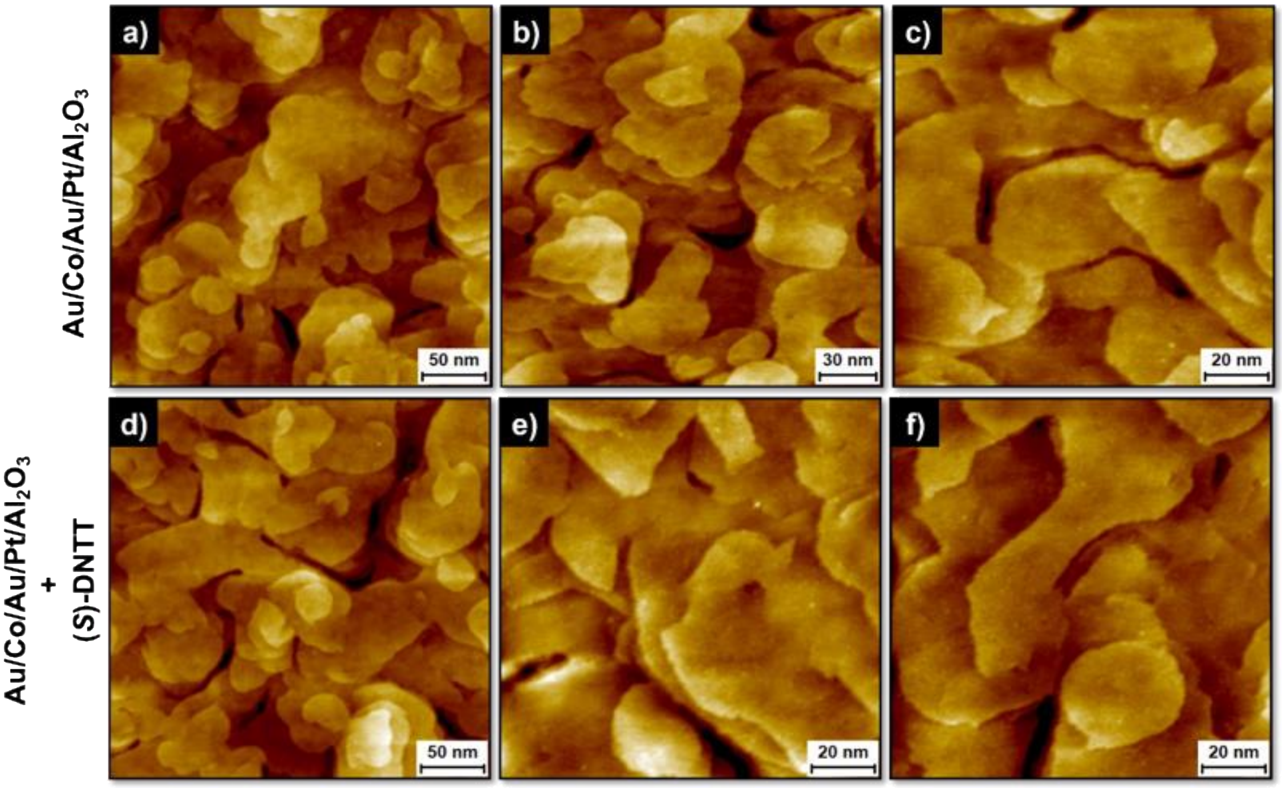

Figure S3: (a-c) STM images of the pristine ferromagnetic substrate (Au/Co/Au/Pt/Al_2O_3) at the air/solid interface. (d-f) 8μM solution of ***(S)*-DNTT** in TCB was drop-casted on the same substrate, and STM measurements were performed at the solution/solid interface. No well-ordered SAMN formation was detected, as evident from the STM images. Imaging conditions: (a) V_{bias} = –0.70 V, I_{set} = 0.10 nA; (b) V_{bias} = –0.70 V, I_{set} = 0.10 nA; c) V_{bias} = –0.70 V, I_{set} = 0.10 nA; (d) V_{bias} = –0.50 V, I_{set} = 0.10 nA; (e) V_{bias} = –0.30 V, I_{set} = 0.10 nA; and (f) V_{bias} = –0.20 V, I_{set} = 0.10 nA.

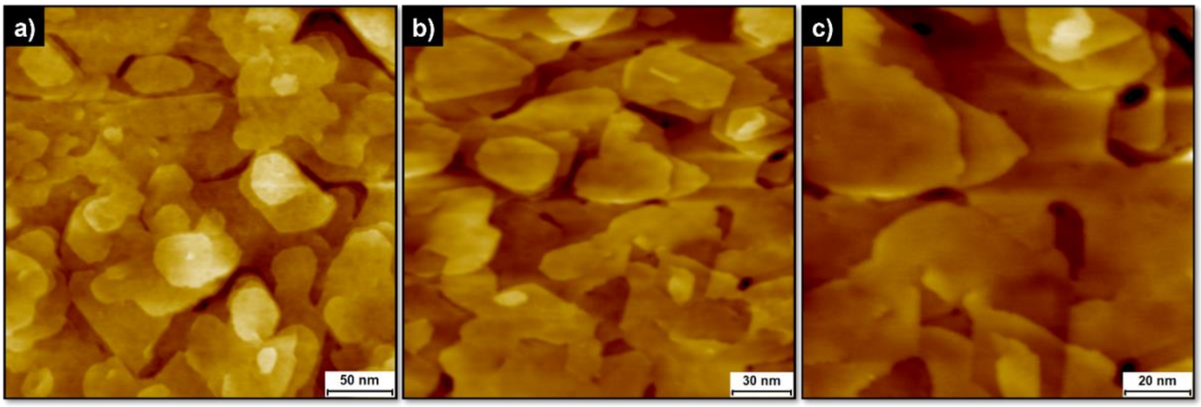

Figure S4: STM images of flame-annealed pristine ferromagnetic (Au/Co/Au/Pt/Al_2O_3) substrate at the air/solid interface. Imaging conditions: (a) V_{bias} = -0.70 V, I_{set} = 0.10 nA; (b) V_{bias} = -0.7 V, I_{set} = 0.05 nA; and c) V_{bias} = -0.7 V, I_{set} = 0.15 nA.

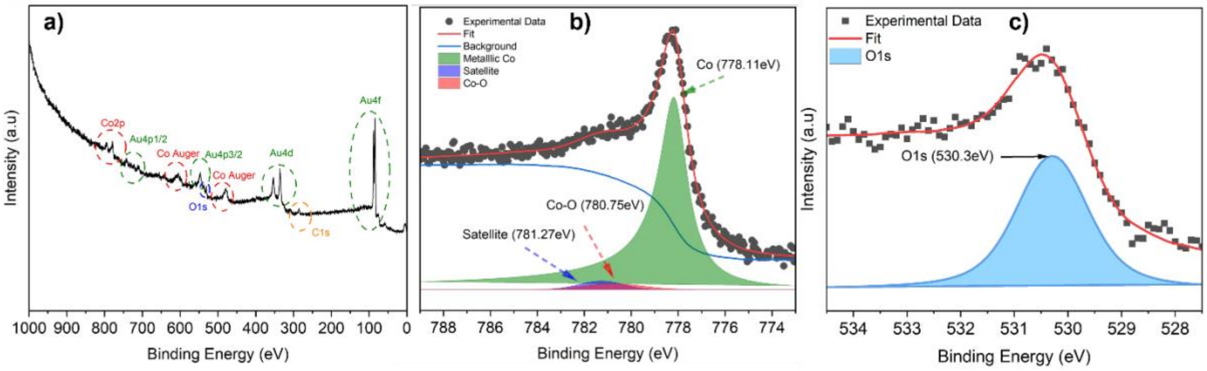

Figure S5: (a) Survey scan of flame annealed ferromagnetic ($Au/Co/Au/Pt/Al_2O_3$) substrate after 30 minutes of sputtering; (b) High-resolution Co 2p3/2 and O1s XPS spectrum of flame annealed ferromagnetic ($Au/Co/Au/Pt/Al_2O_3$) substrate.

Layer	Layer Description	Density	(Lower	Upper)	Thickness	(Lower	Upper)	Roughness	(Lower	Upper)
4, 0	DensityOnly, Au	19.4	(-	-)	5.215	(-	-)	0.446	(-	-)
3, 0	DensityOnly, Co	8.9	(-	-)	1.12	(-	-)	0.52	(-	-)
2, 0	DensityOnly, Au	19.369	(-	-)	19.998	(-	-)	0.466	(-	-)
1, 0	DensityOnly, Pt	21.4	(-	-)	3.767	(-	-)	0.192	(-	-)
Substrate	Sapphire, Al2O3	3.989	(-	-)	600000	(-	-)	0.117	(-	-)

Best fit value: 11.68   Best background: 0.32 counts/s   Best divergence: 0.0164 deg   Best intensity: 61437963 counts/s

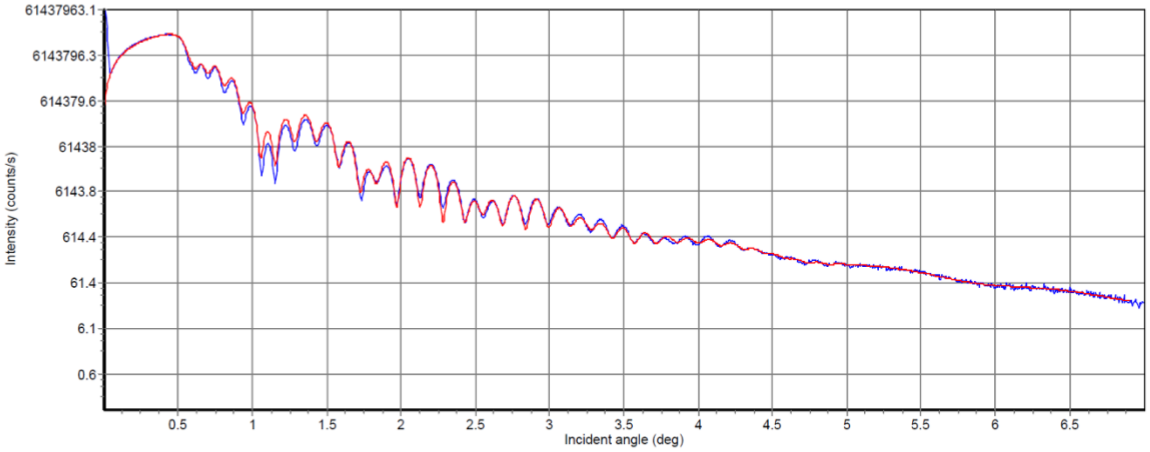

Figure S6: XRR of flame-annealed ferromagnetic ($Au/Co/Au/Pt/Al_2O_3$) substrate. The thickness of the Co layer before and after flame-annealing of ferromagnetic substrates remains almost similar. The thickness of the Co layer before annealing was approximately like original value, *i.e.,* 1.20 nm.

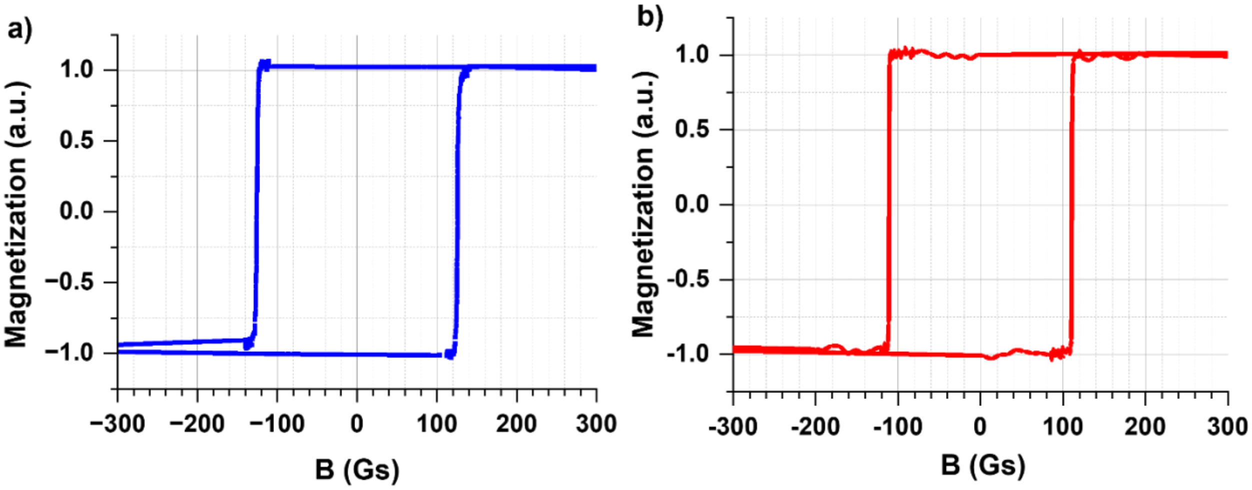

Figure S7: Comparison of P-MOKE of pristine (a) and flame-annealed (b) ferromagnetic ($Au/Co/Au/Pt/Al_2O_3$) substrate. The square shape of the loop with a remanence of about 1, which indicates the existence of a perpendicular anisotropy, remains the same after annealing.

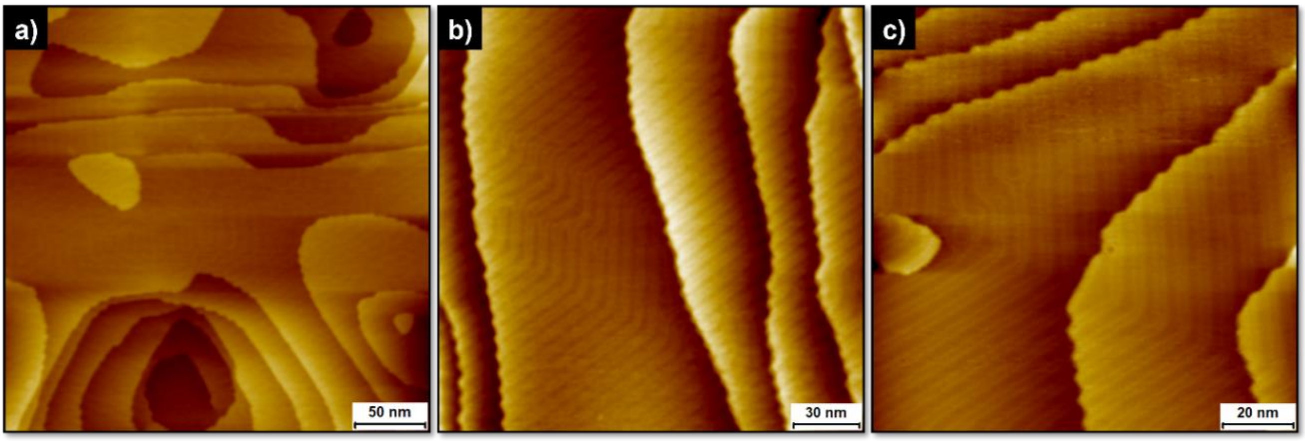

Figure S8: STM images of flame-annealed Au(111) substrate at the solid/air interface. A herringbone reconstruction was observed in STM images of flame-annealed Au surface. Tunneling parameters were: a) V_{bias} = -0.6 V, I_{set} = 0.10 nA; (b) V_{bias} = -0.7 V, I_{set} = 0.10 nA; and (c) V_{bias} = -0.7 V, I_{set} = 0.10 nA.

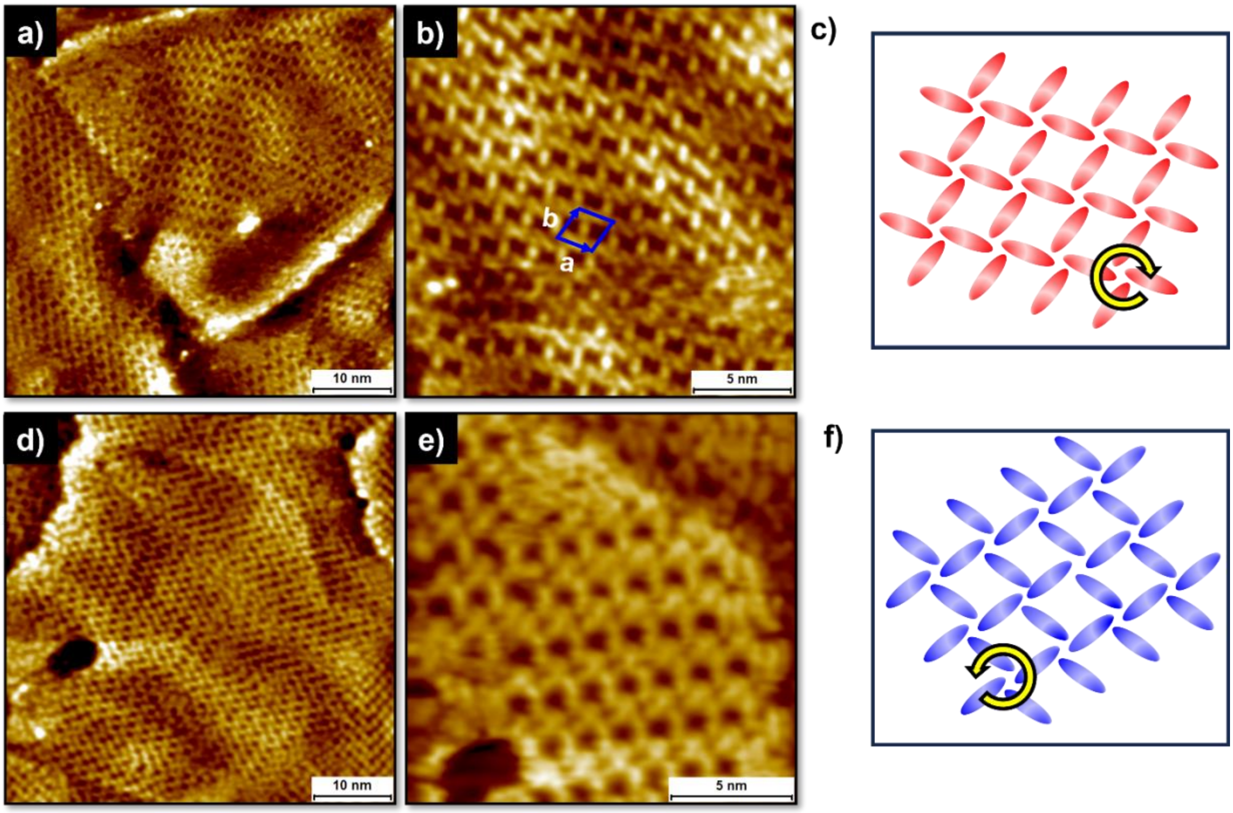

Figure S9: STM images of **(*S*)-DNTT** (a & b) and **(*R*)-DNTT** (d & e) SAMNs at the TCB/ferromagnetic substrate interface; Schematic of molecular arrangement in SAMNs of **(*S*)-DNTT** (c) and **(*R*)-DNTT** (f). Tunneling parameters: (a) V_{bias} = -0.2 V, I_{set} = 0.10 nA; and (b) V_{bias} = -0.2 V, I_{set} = 0.10 nA, (d) V_{bias} = -0.2 V, I_{set} = 0.10 nA; and (e) V_{bias} = -0.19 V, I_{set} = 0.10 nA.

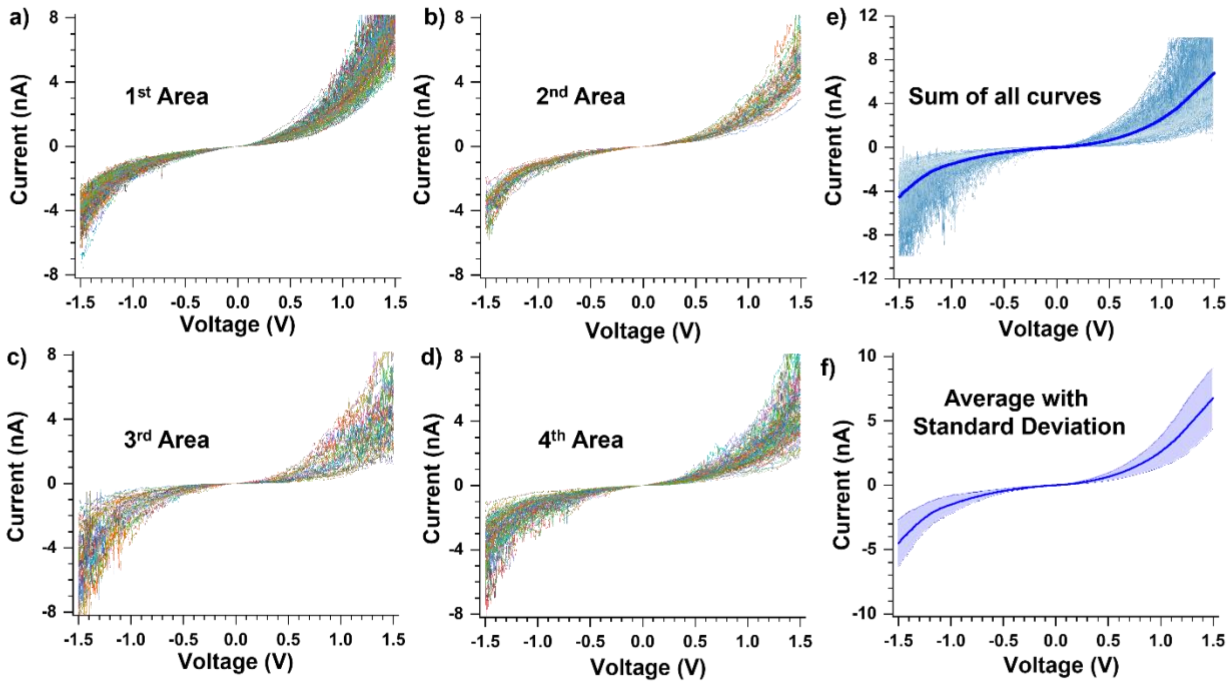

Figure S10: I-V curves of (*S*)-DNTT SAMNs on the ferromagnetic substrate for upward magnetization: (a-d) I-V curves at four different areas of SAMNs of **(*S*)-DNTT** on top of ferromagnetic ($Au/Co/Au/Pt/Al_2O_3$) substrate for upward magnetization direction of Cobalt layer. Different areas were separated by a few 100 nm. All I-V curves were recorded at the solution/solid interfaces. (e) The sum of all I-V curves and the dark blue line represents an averaged value of all I-V curves. (f) Averaged I-V curve with error bar (light blue color area represents standard deviation). The set point was kept at 0.1 nA and bias voltage at -0.2 V.

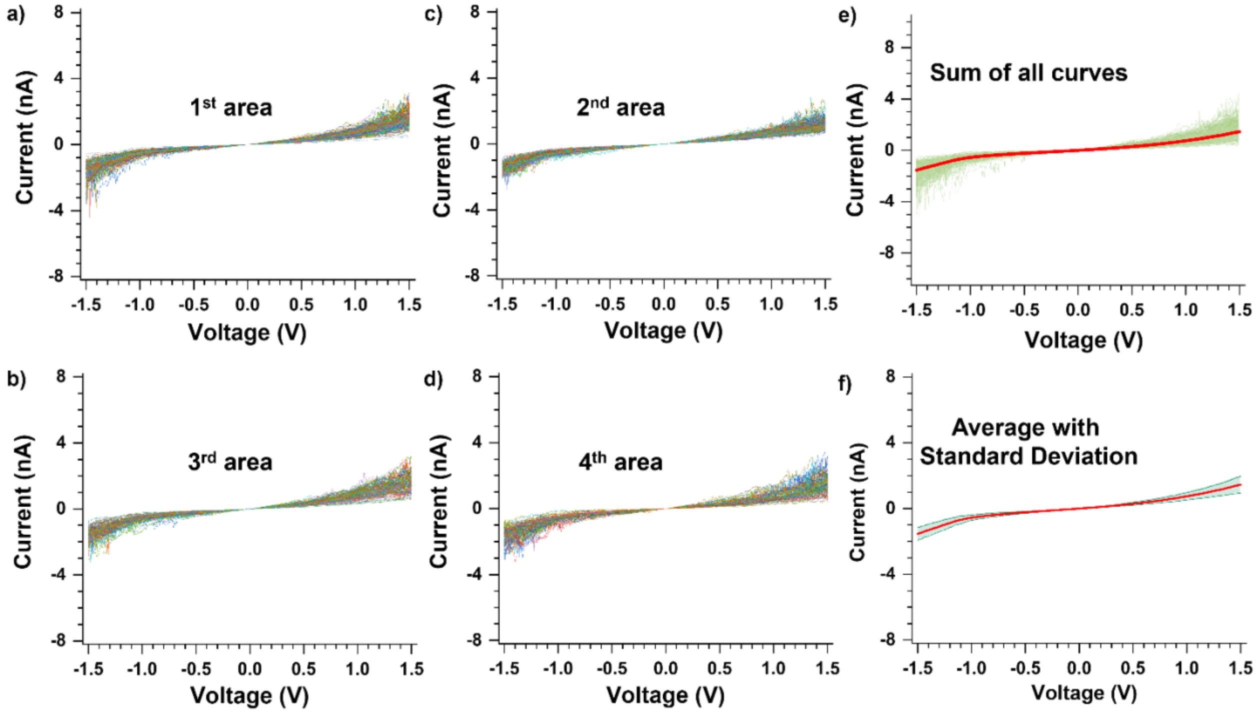

Figure S11: I-V curves of (*S*)-DNTT on the ferromagnetic substrate for downward magnetization: (a-d) I-V curves at four different areas of SAMNs of **(*S*)-DNTT** on top of ferromagnetic (Au/Co/Au/Pt/Al_2O_3) substrate for downward magnetization direction of Cobalt layer. All I-V curves were recorded at the solution/solid interfaces. (e) The sum of all I-V curves and the red line represents the averaged value of all I-V curves. (f) Averaged I-V curve with error bar (light green color area represents standard deviation). The set point was kept at 0.1 nA and bias voltage at -0.2 V.

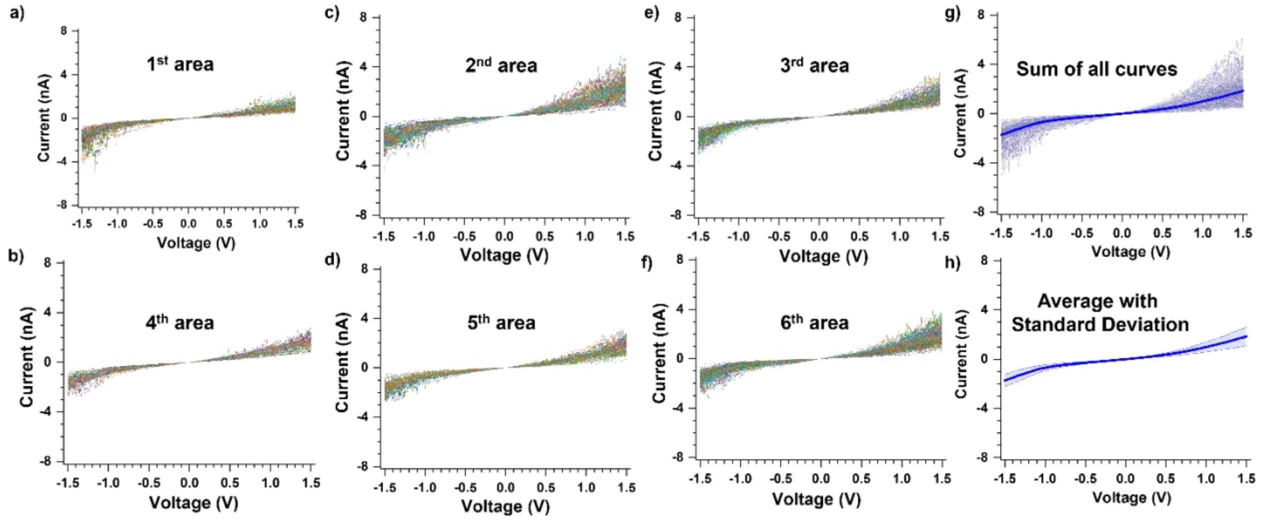

Figure S12: I-V curves of (*R*)-DNTT SAMNs on a ferromagnetic substrate for upward magnetization: (a-f) I-V curves at six different areas across the SAMN of **(*R*)-DNTT** on top of ferromagnetic (Au/Co/Au/Pt/Al_2O_3) substrate for upward magnetization direction of Cobalt layer. (g) The sum of all I-V curves (1st to 6th area) and the blue line represent the averaged value of all I-V curves. (h) Averaged I-V curve with error bar (light blue color area represents standard deviation). All I-V curves were recorded at the solution/solid interfaces. The set point was kept at 0.1 nA and bias voltage at -0.2 V.

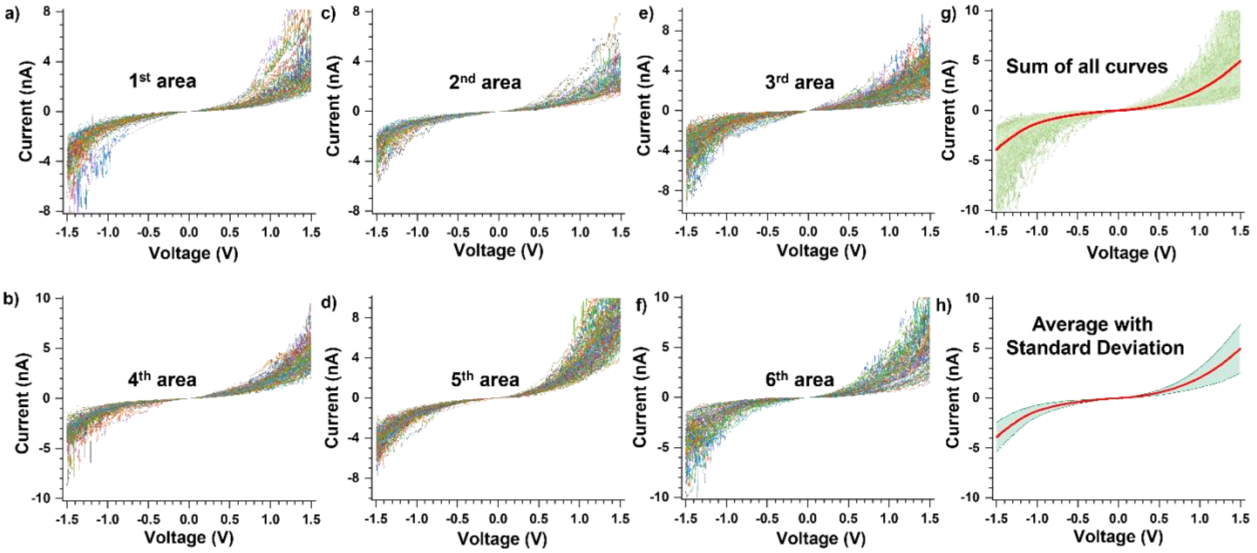

Figure S13: I-V curves of (*R*)-DNTT SAMNs on a ferromagnetic substrate for downward magnetization: (a-f) I-V curves at four different areas of SAMNs of **(*R*)-DNTT** on top of ferromagnetic (Au/Co/Au/Pt/Al_2O_3) substrate for downward magnetization direction of Cobalt layer. (g) The sum of all I-V curves (1st to 6th area) and the blue line represent the averaged value of all I-V curves. (h) Average I-V curve with error bar (light green color area represents standard deviation). All I-V curves were recorded at the solution/solid interfaces. The set point was kept at 0.1 nA and bias voltage at -0.2 V.

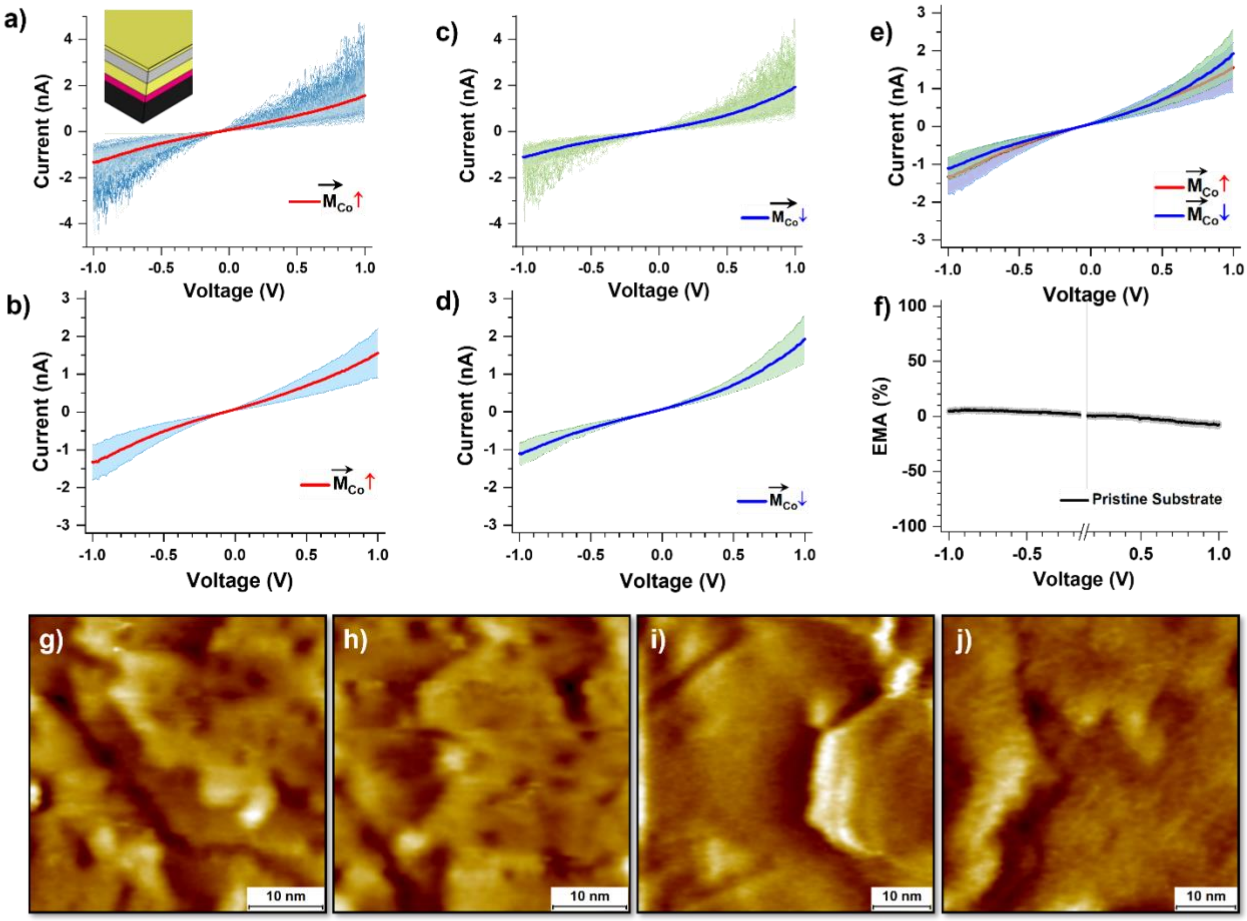

Figure S14: STS on Pristine Substrate: (a-b) STS curves recorded on pristine ferromagnetic (Au/Co/Au/Pt/Al_2O_3) substrate with STM at the air/solid interface for upward magnetization direction of Cobalt layer, red line represents averaged I-V curve and light blue shaded region represents standard deviation in STS curves. (c-d) STS curves recorded on pristine ferromagnetic (Au/Co/Au/Pt/Al_2O_3) substrate with STM at the air/solid interface for downward magnetization direction of Cobalt layer, blue line represents averaged I-V curve and light green shaded region represents standard deviation in STS curves. (e) Comparison of STS curves for upward and downward magnetization direction of Co layer. During STS measurement, the magnet (~250 mT) was kept underneath the ferromagnetic substrate. In each magnetization direction of a magnet, STS curves were recorded at multiple areas. During STS measurements all parameters were kept constant for both magnetization directions. The bias voltage was kept at -0.2 V and a set point at 0.10 nA. (f) EMA for pristine Au/Co/Au/Pt/Al_2O_3 Substrate. (g-j) STM images of a few areas where the above STS curves were recorded. Light grey shaded area represents standard error. Imaging conditions for all STM images are the same *i.e.,* V_{bias} = -0.2 V and I_{set} = 0.10 nA. Non-zero EMA at the ferromagnetic substrate/air interface may results from intrinsic spin-dependent states of the substrate as well as experimental factors such as drift, tip fluctuations, or surface adsorbates.

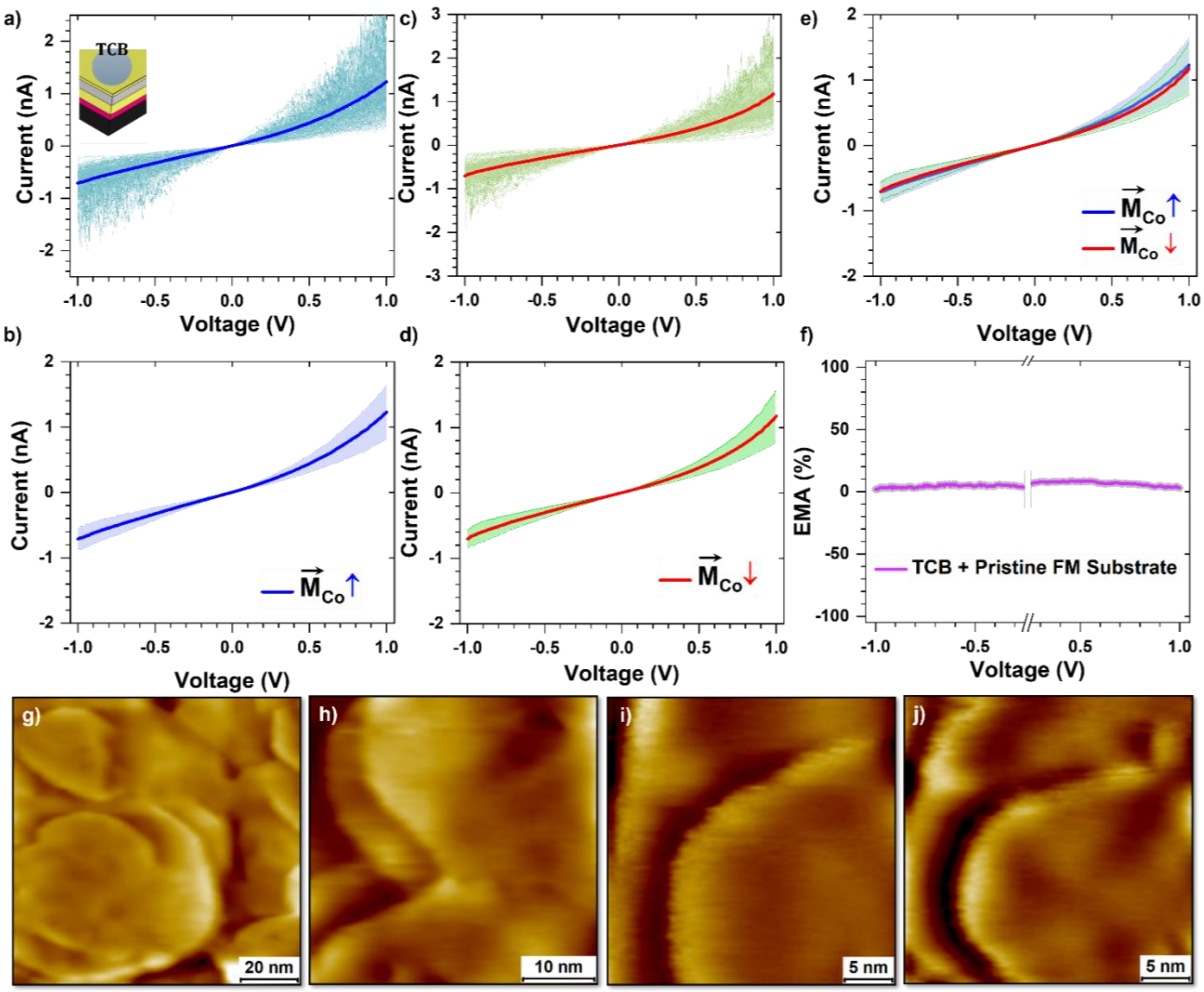

Figure S15: STS on TCB/Pristine Substrate Interface: (a-b) STS curves recorded on pristine ferromagnetic (Au/Co/Au/Pt/Al_2O_3) substrate with STM at the TCB/solid interface for upward magnetization direction of Cobalt layer, red line represents averaged I-V curve, and light blue shaded region represents standard deviation in STS curves. (c-d) STS curves recorded on a pristine ferromagnetic (Au/Co/Au/Pt/Al_2O_3) substrate with STM at the TCB/solid interface for downward magnetization direction of the Cobalt layer, the blue line represents the averaged I-V curve, and the light green shaded region represents the standard deviation in STS curves. (e) Comparison of STS curves for upward and downward magnetization direction of the Co layer in the presence of solvent (TCB). During STS measurement, the magnet (~250 mT) was kept underneath of ferromagnetic substrate. In each magnetization direction of the magnet, STS curves were recorded at multiple areas. During STS measurements, all parameters were kept constant for both magnetization directions. The bias voltage was kept at -0.2 V, and a set point of 0.10 nA. (f) EMA for pristine Au/Co/Au/Pt/Al_2O_3 substrate in the presence of TCB. (g-j) STM images of a few areas where the above STS curves were recorded. Imaging conditions for all STM images are the same, *i.e.*, V_{bias} = -0.2 V and I_{set} = 0.10 nA.

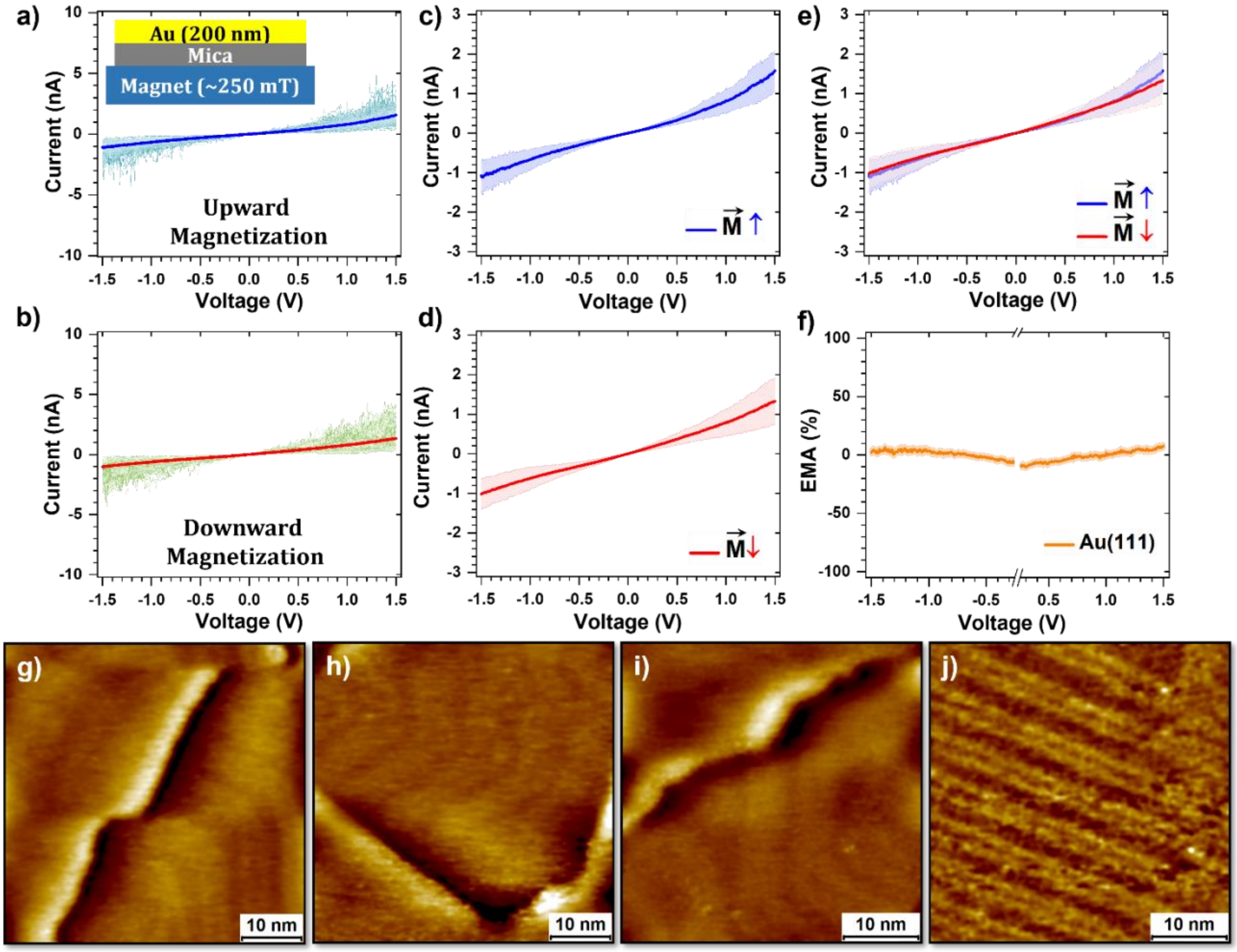

Figure S16: STS on Pristine Au(111)/Mica Substrate: STS curves recorded on pristine Au(111)/Mica substrate with STM at the solid/air interface for upward magnetization (a, b) and downward magnetization (c, d) direction of external magnet. The light blue and light green shaded region represents the standard deviation in STS curves. (e) Comparison of STS curves for upward and downward magnetization direction. (f) EMA for pristine Au(111) substrate. During STS measurement, a magnet (~250 mT) was kept underneath of Au(111)/Mica substrate. STS curves were recorded at multiple areas in each magnetization direction underneath the magnet. During STS measurements, all parameters were kept constant for both magnetization directions. The bias voltage was kept at -0.7 V, and the setpoint at 0.10 nA. (g-j) STM images of a few areas where the above STS curves were recorded. Imaging conditions for all STM images are the same, *i.e.*, V_{bias} = -0.2 V and I_{set} = 0.10 nA.

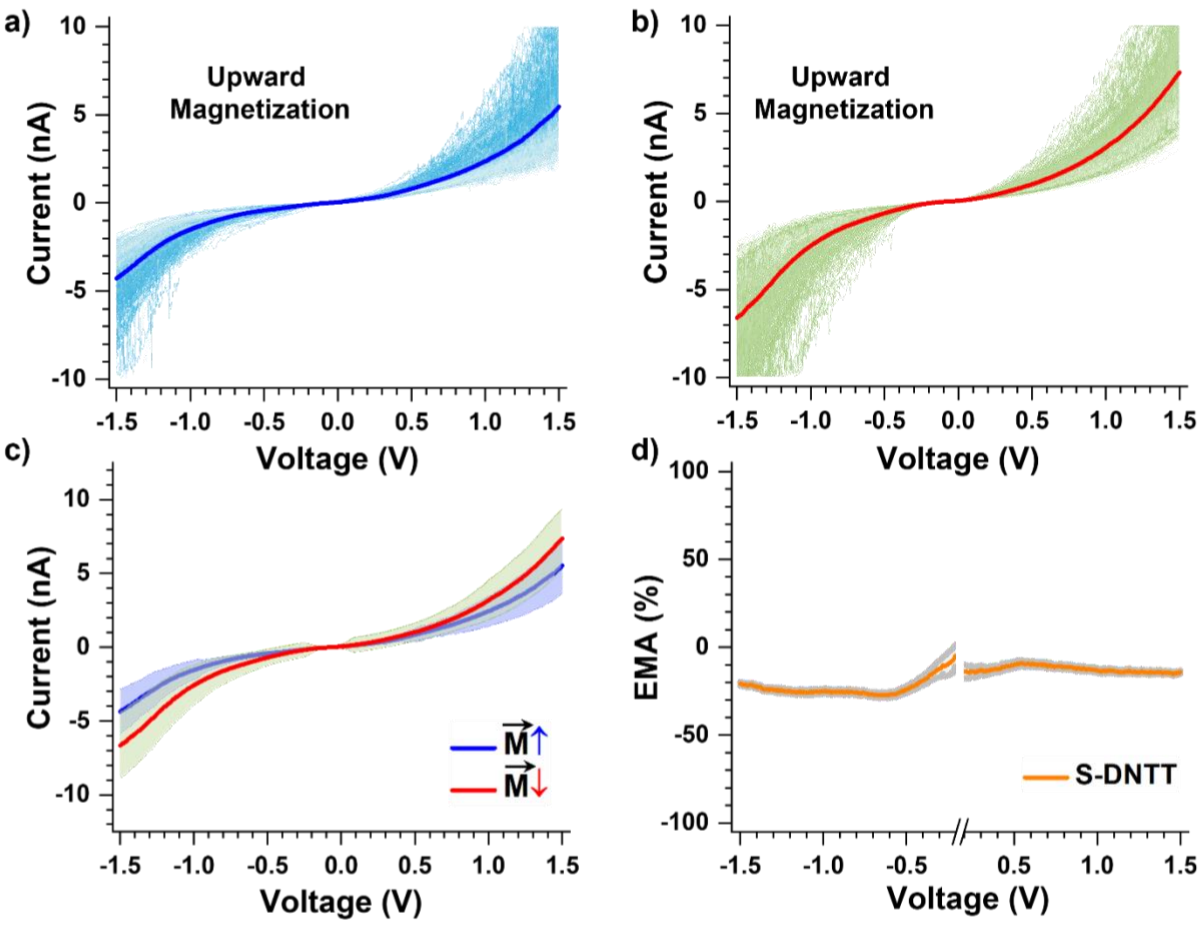

Figure S17: STS on SAMNs of (*S*)-DNTT on Au(111)/Mica Substrate: (a-b) STS curves recorded on SAMNs of **(*S*)-DNTT** on Au(111) surface at the solution/solid interface. During STS measurement, a magnet (~250 mT) was kept underneath of Au(111)/Mica substrate. STS curves were recorded at multiple areas in each magnetization direction underneath the magnet. During STS measurements, all parameters were kept constant for both magnetization directions. The bias voltage was kept at -0.2 V and the set point at 0.10 nA. (c) comparison of averaged I-V curves for upward and downward magnetization direction of the external magnet. The light blue and green shaded area represents the standard deviation. (d) EMA for **(*S*)-DNTT** at the solution/solid interface. The non-zero EMA in SAMNs of S-DNTT on Au(111)/mica likely reflects a combination of proximity-induced effects in Au from the external magnet, intrinsic CISS filtering by the chiral molecules, and experimental asymmetries in the STM junction.

S2: Ab-initio Modelling

Computationally efficient Tight Binding (TB) calculations were used to extensively explore the Potential Energy Surface and pre-optimize structures of all investigated systems. For this, the GFN1-xTB Hamiltonian[5] was used within the DFTB+ program.[6]

DFT calculations were performed with the PBE functional[7] augmented by Many Body Dispersion corrections [7, 8] implemented in the Fritz Haber Institute ab initio molecular simulations (FHI-aims)[9-12] package using "light" or "light_194" numerical orbitals. Zeroth Order Regular Approximation (ZORA) was utilized to account for relativistic effects. Also, some of the optimizations were performed within the Atomic Simulation Environment (ASE).

The Au (111) surface was modelled as a slab with 4 Au layers saturated with hydrogen atoms on one side, Figure S18, as this yielded a good compromise between size and accuracy. On this slab, a comprehensive exploration for the different adsorption modes of a simplified molecular analogue, **dimethylated-DNTT (Me-DNTT)**, were performed. A model to reproduce the experimental SAMNs, Figure S20, was built based on the two lowest energy adsorption modes of Me-DNTT, with binding energies of -2.341 eV and -2.238 eV, Figure S20, top and bottom, respectively.

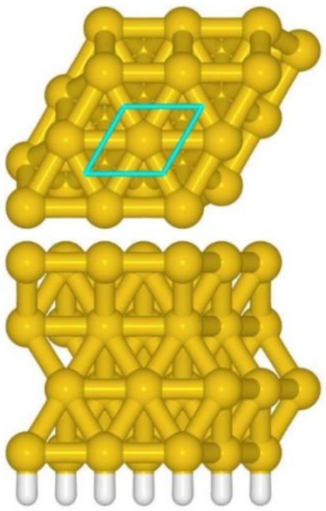

Figure S18: Hydrogen terminated 4-layer slab used to model the Au (111) surface.

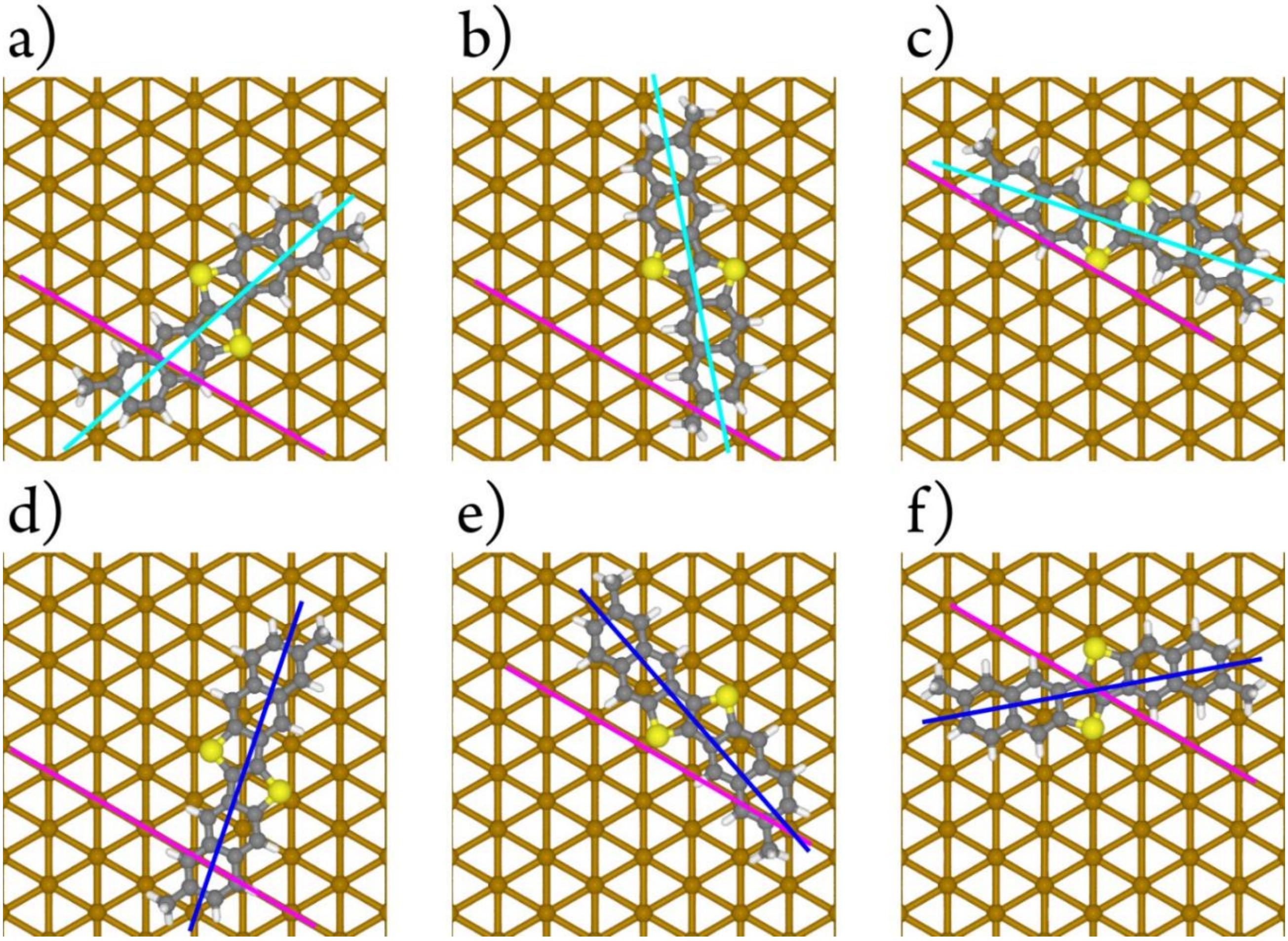

Figure S19: First, (a-c), and second, (d-e), adsorption modes of **Me-DNTT** on gold (111) in the three possible orientations equivalent by symmetry. Purple lines indicate the (110) direction in the surface unit cell while blue lines highlight the long molecular axis of **DNTT.**

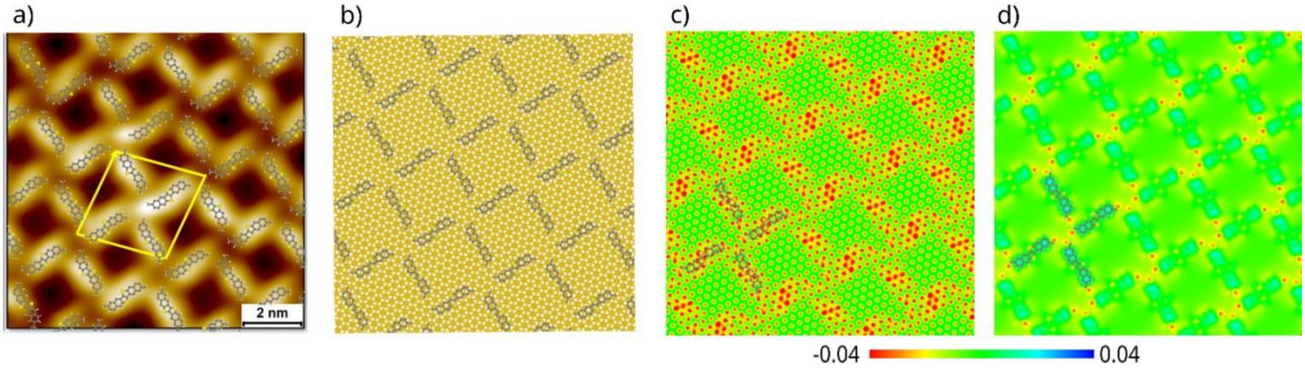

Figure S20: Experimental STM image of ***(R)*-DNTT** SAMN with overlay of DFT model built with **Me-DNTT** (a). Full DFT self-assembled molecular network on gold (111) surface model (b). Corresponding electrostatic potential at a plane between the surface and the adsorbed molecules (c), electrostatic potential with the gold surface removed (d), all values in atomic units.

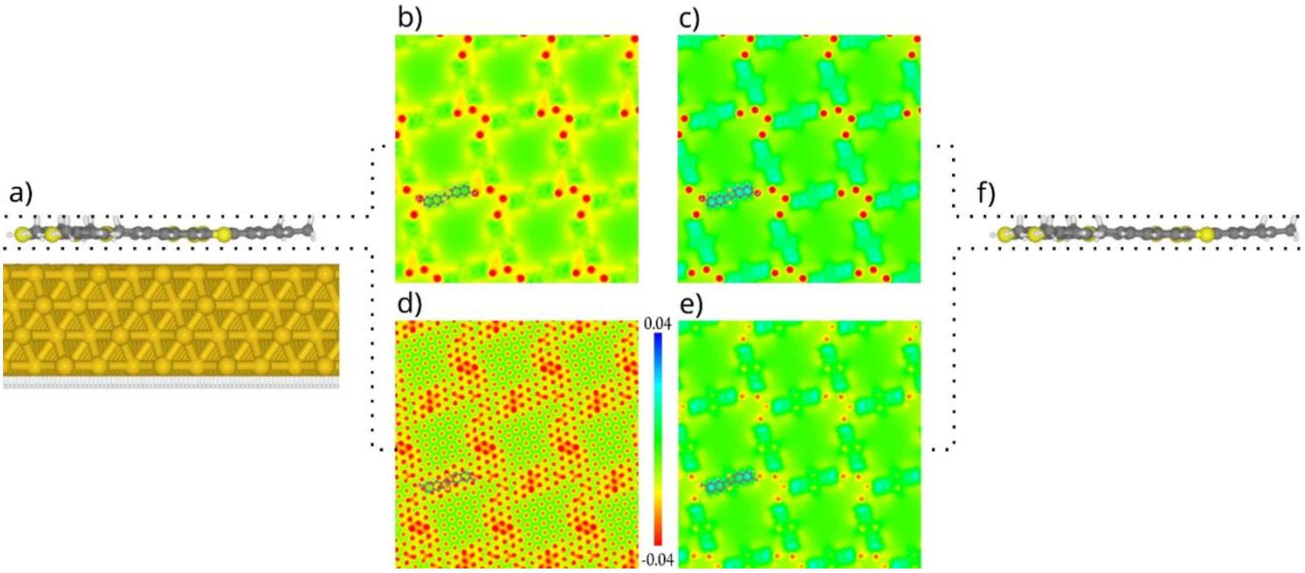

Figure S21: Electrostatic potentials of the DFT model of the self-assembled molecular network of **Me-DNTT** on a gold surface (b, d) and self-standing (c, e). Scheme showing the planes of measurement for both, on gold surface and self-standing systems, is shown on panels a) and f) accordingly. As a reference, an adsorbed molecule is rendered in panels (b-e). All values in atomic units.

References:

(1) Volpi, M.; Jouclas, R.; Liu, J.; Liu, G.; Catalano, L.; McIntosh, N.; Bardini, M.; Gatsios, C.; Modesti, F.; Turetta, N.; et al. Enantiopure Dinaphtho[2,3-b:2,3-f]thieno[3,2-b]thiophenes: Reaching High Magnetoresistance Effect in OFETs. *Adv. Sci.* **2023**, *10* (26), 2301914.

(2) Lee, H.-B.-R.; Kim, H. High-Quality Cobalt Thin Films by Plasma-Enhanced Atomic Layer Deposition. Electrochem. *Solid-State Lett.* **2006**, 9 (11), G323.

(3) Klebanoff, L. E.; Van Campen, D. G.; Pouliot, R. J. Spin-Resolved and High-Energy-Resolution XPS Studies of Cobalt Metal and a Cobalt Magnetic Glass. *Phys. Rev. B* **1994**, 49 (3), 2047–2057.

(4) McIntyre, N. S.; Johnston, D. D.; Coatsworth, L. L.; Davidson, R. D.; Brown, J. R. X-ray Photoelectron Spectroscopic Studies of Thin Film Oxides of Cobalt and Molybdenum. *Surf. Interface Anal.* **1990**, 15 (4), 265–272.

(5) Grimme, S.; Bannwarth, C.; Shushkov, P. A Robust and Accurate Tight-Binding Quantum Chemical Method for Structures, Vibrational Frequencies, and Noncovalent Interactions of Large Molecular Systems Parametrized for All spd-Block Elements (Z = 1–86). *J. Chem. Theory Comput.* **2017**, *13* (5), 1989-2009.

(6) Aradi, B.; Hourahine, B.; Frauenheim, T. DFTB+, a Sparse Matrix-Based Implementation of the DFTB Method. *J. Phys. Chem. A* **2007**, *111* (26), 5678-5684.

(7) Perdew, J. P.; Burke, K.; Ernzerhof, M. Generalized Gradient Approximation Made Simple. *Phys. Rev. Lett.* **1996**, *77* (18), 3865-3868.

(8) Ambrosetti, A.; Reilly, A. M.; DiStasio, R. A., Jr.; Tkatchenko, A. Long-range correlation energy calculated from coupled atomic response functions. *J. Chem. Phys.* **2014**, *140* (18).
(9) Blum, V.; Gehrke, R.; Hanke, F.; Havu, P.; Havu, V.; Ren, X.; Reuter, K.; Scheffler, M. Ab initio molecular simulations with numeric atom-centered orbitals. *Comput. Phys. Commun.* **2009**, *180* (11), 2175-2196.
(10) Marek, A.; Blum, V.; Johanni, R.; Havu, V.; Lang, B.; Auckenthaler, T.; Heinecke, A.; Bungartz, H. J.; Lederer, H. The ELPA library: scalable parallel eigenvalue solutions for electronic structure theory and computational science. *J. Phys.: Condens. Matter* **2014**, *26* (21), 213201.
(11) Yu, V. W.-z.; Corsetti, F.; García, A.; Huhn, W. P.; Jacquelin, M.; Jia, W.; Lange, B.; Lin, L.; Lu, J.; Mi, W.; et al. ELSI: A unified software interface for Kohn–Sham electronic structure solvers. *Comput. Phys. Commun.* **2018**, *222*, 267-285.
(12) Havu, V.; Blum, V.; Havu, P.; Scheffler, M. Efficient O(N) integration for all-electron electronic structure calculation using numeric basis functions. *J. Comput. Phys.* **2009**, *228*, 8367-8379.
